# Palladium-catalyzed synthesis and nucleotide pyrophosphatase inhibition of benzo[4,5]furo[3,2-*b*]indoles

**DOI:** 10.3762/bjoc.15.276

**Published:** 2019-11-22

**Authors:** Hoang Huy Do, Saif Ullah, Alexander Villinger, Joanna Lecka, Jean Sévigny, Peter Ehlers, Jamshed Iqbal, Peter Langer

**Affiliations:** 1 Institut für Chemie, Universität Rostock, Albert Einstein Str. 3a, 18059 Rostock, Germany; 2 Faculty of Chemistry, VNU Hanoi University of Science, 19 Le Thanh Tong, Hoan Kiem, Hanoi, 110403, Vietnam; 3 Centre for Advanced Drug Research, COMSATS University Islamabad, Abbottabad Campus, Abbottabad-22060, Pakistan; 4 Département de microbiologie-infectiologie et d'immunologie, Faculté de Médecine, Université Laval, Québec, QC, G1V 0A6, Canada; 5 Centre de Recherche du CHU de Québec – Université Laval, Québec, QC, G1V 4G2, Canada; 6 Leibniz-Institut für Katalyse an der Universität Rostock e.V., Albert Einstein Str. 29a, 18059 Rostock, Germany

**Keywords:** Buchwald–Hartwig reaction, cyclization, N-heterocycles, palladium, Suzuki–Miyaura reaction

## Abstract

A two-step palladium-catalyzed procedure based on Suzuki–Miyaura cross coupling, followed by a double Buchwald–Hartwig reaction, allows for the synthesis of pharmaceutically relevant benzo[4,5]furo[3,2*-b*]indoles in moderate to very good yield. The synthesized compounds have been analyzed with regard to their inhibitory activity (IC_50_) of nucleotide pyrophosphatases *h*-NPP1 and *h*-NPP3. The activity lies in the nanomolar range. The results were rationalized based on docking studies.

## Introduction

**Figure 1:**
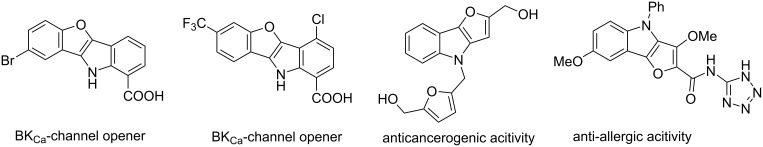
Pharmacologically relevant furoindoles.

Furoindoles and their derivatives have received a lot of attention based on their versatile pharmaceutical activities. Furoindols were reported to show potent antiallergic [1–4], anticancer [5–6], analgesic and anti-inflammatory activity [7] (Figure 1). Similarly, benzofuroindol has been studied intensively as a pharmacophore of calcium-activated potassium channel (BK_Ca_) opening activity [8–13]. Hence, benzofuroindols have been discussed as potential drug candidates for antispasmodic activity and thus therapeutic treatment of, e.g., urge urinary incontinence [8–9,11]. In addition, derivatives might be applied for therapeutic treatment of stroke, asthma, hypertensions, convulsion, traumatic brain injury [10] or treatment of erectile dysfunction [12–13]. Other derivatives show selective activity as estrogen, androgen and/or progestrin receptor modulators [14].

Hence, the synthesis of furoindoles has been intensively studied in recent years [8,10,15–21]. We and others extensively studied double Buchwald–Hartwig reactions as the key step for the synthesis of heterocycles. For example, the cyclization of 2,2’-dibromobiaryls with amines allows for a convenient synthesis of carbazole derivatives [22–30]. Recently, we reported the synthesis of diindolofurans by regioselective Suzuki–Miyaura couplings of tetrabromofuran and subsequent cyclization by tetrafold Buchwald–Hartwig reaction [31]. We also studied the synthesis of benzo[4,5]furo[3,2-*b*]indoles by a similar concept. However, while performing our studies, Truong et al. reported the synthesis of these target molecules by a related strategy. The cyclization of 2-alkynylphenols with iodinde gave a 2-(2-bromophenyl)-3-iodobenzo[*b*]furan which could be cyclized by Buchwald–Hartwig reactions [32]. Altogether, the synthesis of four derivatives was reported. Herein, we wish to report the synthesis of ten benzo[4,5]furo[3,2-*b*]indole derivatives based on regioselective Suzuki–Miyaura reaction of 2,3-dibromobenzofuran with 2-bromophenylboronic acid and subsequent cyclization. The difference between the work of Truong and our approach mainly lies in the synthesis of the cyclization precursor. The method of Truong and our approach are equally efficient in this regard. However, the use of 2-alkynylphenols, as reported by Truong, requires one or two additional synthetic steps. The Buchwald–Hartwig reaction was individually optimized by both groups. While Truong and co-workers exclusively used anilines as reagents, we also successfully employed alkyl- and benzylamines which required an additional optimization of the conditions and the employment of different ligands. Therefore, we feel that our approach is more general and merits publication. In addition to the synthetic work, we report, for the first time, a study related to the activity of the products as nucleotide pyrophosphatase inhibitors. In this context, we also studied the biological activity of previously synthesized diindolofurans and the results are compared with those of benzofuroindoles.

## Results and Discussion

**Scheme 1:**
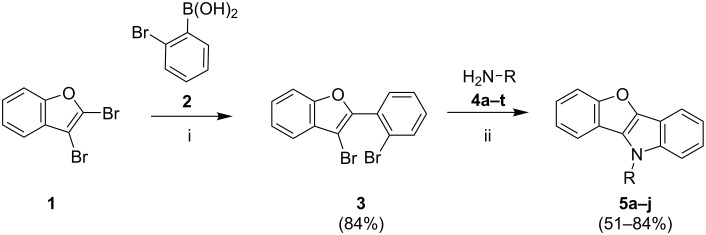
Synthesis of benzo[4,5]furo[3,2-*b*]indoles **5a–j**. Conditions: (i) 1.2 equiv 2-bromophenylboronic acid (**2**), 5 mol % Pd(PPh_3_)_4_, 3.0 equiv K_3_PO_4_, 1,4-dioxane, H_2_O, 100 ºC, 8 h. (ii) 1.5 equiv **4**, 3.0 equiv NaO*t-*Bu, 5 mol % Pd_2_(dba)_3_, 10 mol % ligand, toluene, 110 ºC, 12 h.

Following a literature procedure, 2,3-dibromobenzofuran (**1**) was synthesized by bromination of benzofuran [33]. The Suzuki–Miyaura reaction of **1** with 2-bromophenylboronic acid (**2**), carried out under standard conditions using Pd(PPh_3_)_4_, afforded the desired product **3** in 84% yield and with very good regioselectivity. The synthesis of benzo[4,5]furo[3,2-*b*]indoles by double Buchwald–Hartwig reaction was studied next. The conditions were optimized for the reaction of **3** with *p*-toluidine (**4b**, Scheme 1, Table 1). The amount of ligand and palladium precursor was optimized using different solvents (dioxane, toluene, and DMF). Monodentate ligands, like XPhos, SPhos, DavePhos, RuPhos, or P(*t-*Bu)_3_·HBF_4_, were not effective in the reaction and gave product **5b** in low yields. Bidentate phosphine ligands, such as BINAP, XantPhos, dppe, or dppf (Table 1), worked very well and allowed to improve the yield of **5b** up to 75% (Table 1, entry 4). As compared to Pd_2_(dba)_3_, the use of Pd(OAc)_2_ as the Pd source resulted in a decrease of the yield (52%). Performing the reaction in dioxane or DMF gave lower yields as well. In summary, up to 75% yield of **5b** could be achieved using BINAP and Pd_2_(dba)_3_ as the catalytic system.

**Table 1:** Optimization for the Synthesis of **5b**.

Entry	Pd precursor	Ligand	Yield (%)^a^

**1**	Pd_2_(dba)_3_	dppf	57
**2**	Pd_2_(dba)_3_	XantPhos	54
**3**	Pd_2_(dba)_3_	dppe	62
**4**	Pd_2_(dba)_3_	BINAP	75
**5**	Pd_2_(dba)_3_	XPhos	36
**6**	Pd_2_(dba)_3_	SPhos	44
**7**	Pd_2_(dba)_3_	DavePhos	15
**8**	Pd_2_(dba)_3_	RuPhos	45
**9**	Pd_2_(dba)_3_	P*t-*Bu_3_·HBF_4_	41
**10**	Pd(OAc)_2_	BINAP	52
**11**^b^	Pd_2_(dba)_3_	BINAP	61
**12**^c^	Pd_2_(dba)_3_	BINAP	14

^a^Yield calculated by ^1^H NMR of the crude product using 1,4-dioxane as an internal standard. ^b^Dioxane. ^c^DMF.

Subsequently, the scope of the reaction of **3** was studied using different amines. The reaction of **3** with various anilines afforded products **5a–g** in good to excellent yields (Table 2). No impact of the functional groups of the anilines on the yield was observed. The reactions of **3** with aliphatic or benzylic amines under our optimized conditions proved to be unsuccessful and resulted in the formation of complex mixtures. Therefore, an additional ligand optimization was carried out for the reaction of **3** with benzylamine. It was found that DavePhos in the presence of Pd_2_dba_3_ improved the yield of product **5h** drastically to 67% (Table 2). Consequently, our newly optimized conditions allowed the synthesis of products **5h–j** in moderate to good yields (Table 2).

**Table 2:** Synthesis of **5a–j**^a^.

**5**	R	Yield (%)^b^

**a**	Ph	63
**b**	4-MeC_6_H_4_	75
**c**	4-FC_6_H_4_	79
**d**	3-(CF_3_)C_6_H_4_	81
**e**	4-(MeO)C_6_H_4_	65
**f**	3,4-(MeO)_2_C_6_H_3_	51
**g**	4-*t-*BuC_6_H_4_	84
**h**	Bn	67^c^
**i**	*n*-C_7_H_15_	53^c^
**j**	Cyclohexyl	57^c^

^a^Conditions: 1.5 equiv amine **4**, 3.0 equiv NaO*t-*Bu, 5 mol % Pd_2_(dba)_3_, 10 mol % ligand, toluene, 110 ºC, 12 h; ^b^Isolated yields; ^c^DavePhos as ligand.

**Figure 2:**
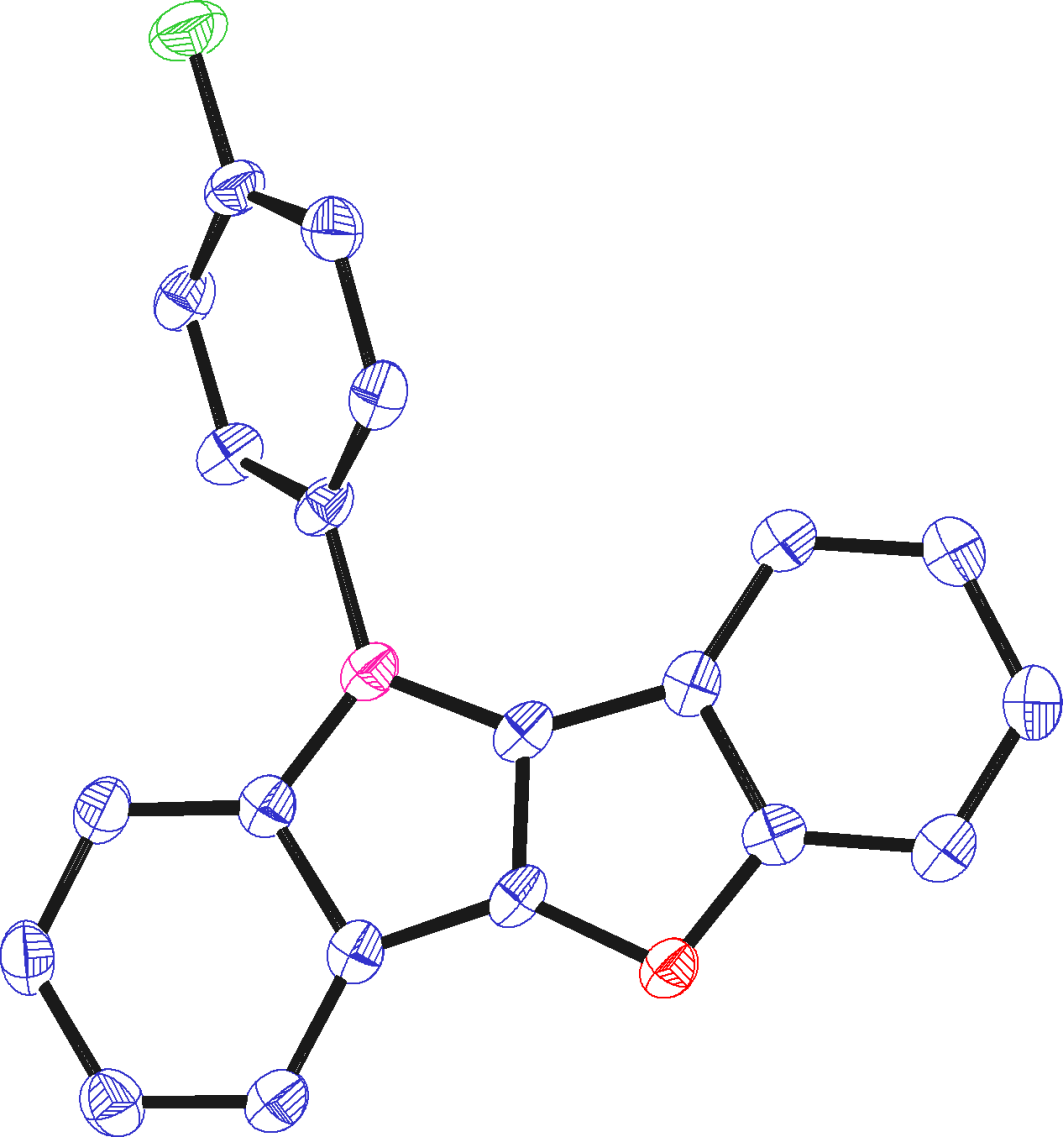
Ortep of **5c** (propability of ellipsoids: 45%).

The structure of **5c** was independently confirmed by X-ray crystal structure analysis. Figure 2 shows the planar benzofuroindol core structure with an orthogonally oriented aryl substituent located on the nitrogen atom.

### Nucleotide pyrophosphatase activity

**Figure 3:**
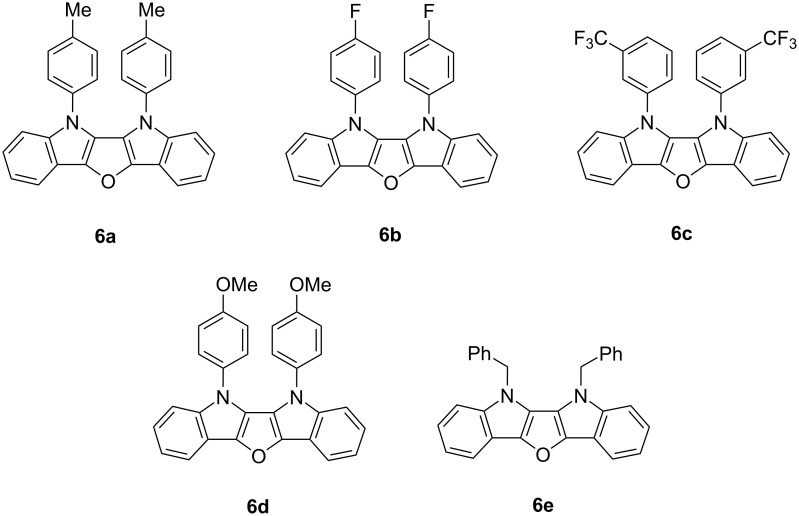
Diindolofurans **6a–e**.

Nucleotide pyrophosphatases belong to the family of ecto-nucleotidases [34–35]. They can hydrolyze nucleotides, dinucleotides, and nucleotide sugars, e.g., ATP, ADP, NAD^+^, ADP-ribose and diadenosine polyphosphates [36]. The isozymes of NPPs are involved in the pathophysiology of different diseases, such as calcification, cancer, and insulin resistance [34–35]. All derivatives of compounds **5** were tested for human recombinant NPPs, i.e., NPP1–3. In addition, we compared the obtained results with those of recently reported diindolofurans **6a–e** (Figure 3) [31]. All compounds show significant inhibition of enzyme *h*-NPP-3 (Table 3) and most of them of the enzyme *h*-NPP-1.

**Table 3:** Biological activity of **5** and **6**.

	*h*-NPP-1 IC_50_ (µM) ± SEM^a^	*h*-NPP-3 IC_50_(µM) ± SEM^a^

**5a**	–	1.38 ± 0.03
**5b**	2.84 ± 0.06	0.59 ± 0.02
**5c**	1.29 ± 0.07	3.14 ± 0.09
**5d**	3.57 ± 0.03	0.49 ± 0.04
**5e**	–	0.26 ± 0.01
**5h**	2.62 ± 0.03	0.27 ± 0.06
**5i**	3.27 ± 0.08	2.55 ± 0.07
**5j**	6.14 ± 0.09	2.39 ± 0.05
**6a**	0.11 ± 0.06	0.61 ± 0.09
**6b**	–	0.13 ± 0.06
**6c**	–	0.28 ± 0.04
**6d**	1.38 ± 0.09	0.18 ± 0.01
**6e**	0.53 ± 0.09	0.21 ± 0.04

^a^The IC_50_ is the concentration at which 50% of the enzyme activity is inhibited.

Compound **5a**, containing a phenyl substituent, and compound **5e,** containing a *p*-methoxyphenyl group, showed a selective inhibitory response towards nucleotide pyrophosphatase enzyme h-NPP-3. In case of **5e**, an inhibitory value IC_50_ ± SEM = 0.26 ± 0.01 µM was observed which, thus, might be considered as potential inhibitor of *h*-NPP-3. Compound **5c** with an inhibitory value for *h*-NPP-1 of IC_50_ ± SEM = 1.29 ± 0.07 µM was more active against NPP1 than against NPP3, while compounds **5b**, **5d**, and **5h** were more active against NPP3 than NPP1. Compounds **5j** and **5i**, containing aliphatic groups, were found to be much less active against both of the two enzymes.

Compounds **6b** and **6c** with fluorinated functional groups (F-C_6_H_4_ and CF_3_C_6_H_4_) proved to be highly selective towards *h*-NPP-3 (IC_50_ ± SEM = 0.13 ± 0.06 and 0.28 ± 0.04, respectively). In fact, they had no interaction with enzyme *h*-NPP-1. Compound **6a**, containing a tolyl group, was active against both *h*-NPP-1 and *h*-NPP-3, but was more selective to *h*-NPP-1 with an inhibitory value of IC_50_ ± SEM = 0.11 ± 0.06 µM. In contrast, compounds **6d** with a methoxy group and **6e** with a benzyl group were more active against NPP-3 than against NPP-1.

Compounds **5e** and **6d** with the methoxy functional group showed high inhibition of *h*-NPP-3. This suggests that the methoxy group could enhance the inhibition of *h*-NPP-3. Furo[3,2-*b*,4,5-*b*']diindoles **6** exhibited an even stronger activity than derivatives **5** which might be caused by their bigger heterocyclic moiety. All compounds **5** and **6** were active to inhibit enzymes *h-*NPP-3, which suggests that the furoindole core structure is the main pharmacophore for the inhibition against *h*-NPP, while changes of the substitution pattern allow for a modification of the selectivity and activity of these compounds to these enzymes.

### Docking studies of *h*-ENPP1 inhibitors

**Figure 4:**
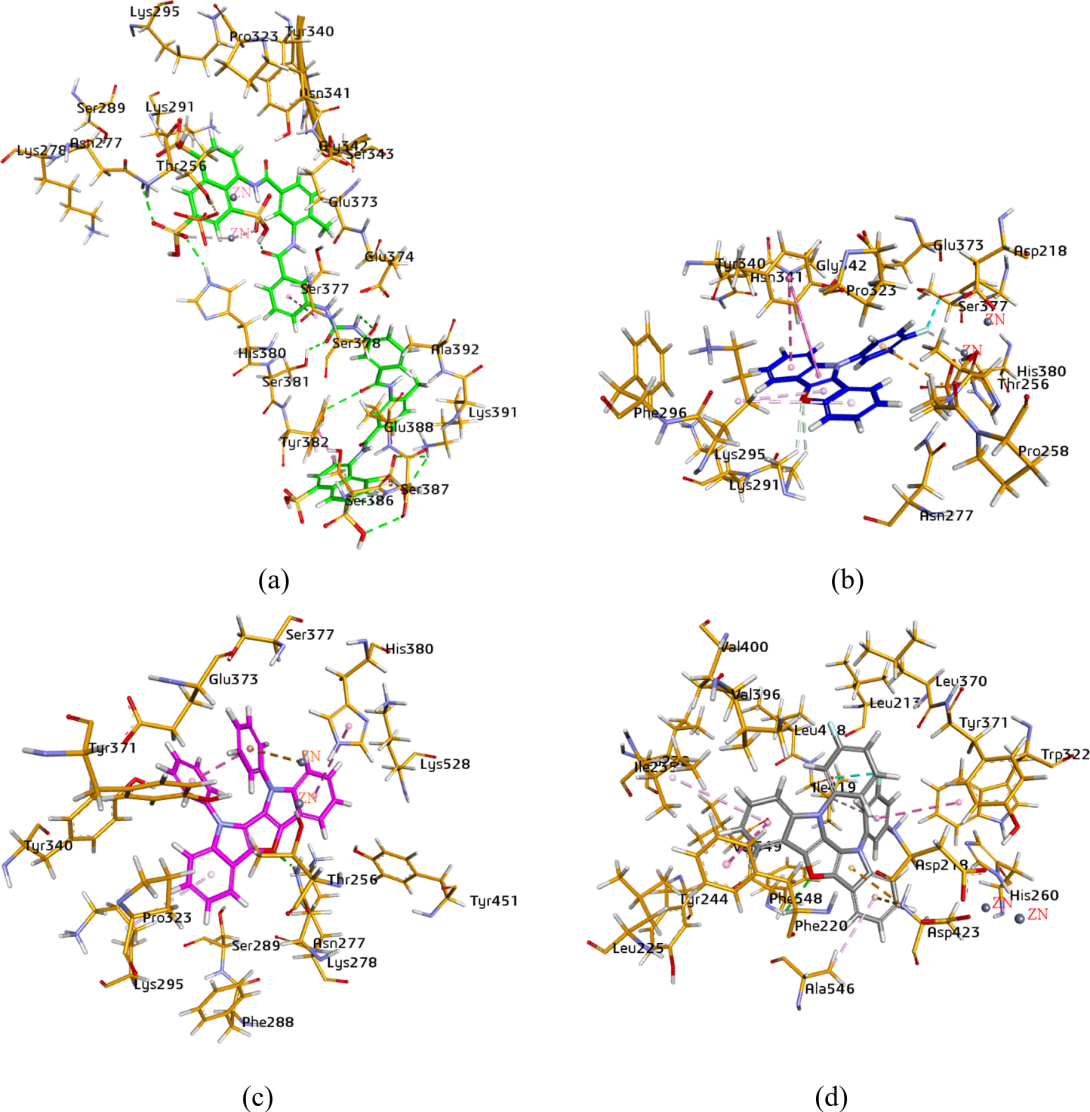
Illustration of binding poses of selected inhibitors for the ENPP1 homology model: (a): suramin, (b): **5c**, (c): **6a**, (d): **6e**.

Molecular docking of the most potent compounds **5c** and **6a** (for ENPP1) and for **6e** (exhibiting dual inhibition for both isozymes) were performed to identify binding interactions, as illustrated in Figure 4. The 3D binding interaction study of suramin revealed a number of binding interactions with amino acid residues. Due to its bulky structure, suramin showed linkage inside and over the surface of the enzyme pocket. The important residues involved in hydrogen bonding were His380, Asn277, Ser378, Ser381, Tyr382, Lys391, Ser387 and Ser386. In addition, the study also showed binding with the zinc ion and π-interactions, in particular, π–π T-shaped and amide–π shaped coupling with Ser377 and Tyr382. The molecular docking study of compound **5c** exhibited π–π T-shaped interactions connecting the indole and furan rings with Tyr340. However, π–alkyl linkage was observed between the benzofuran moiety of compound **5c** and amino acid Lys295. The fluorine atom of the 4-fluorophenyl group was involved in the binding with the zinc ion and Ser377. Hydrogen bonding was found between the oxygen of the benzofuran ring and the hydrogens of Lys291. When compound **6a** was docked inside the active pocket of the homology model, it represented π–π stacked and π–alkyl attachment of the indole rings on both sides of the molecule with amino acids His380 and Lys295. However, the zinc–metal interaction was exhibited by the substituted phenyl moiety on the indole ring. The oxygen atom of the furan ring of dual inhibitor **6e** showed a single hydrogen bond with Phe548. Asp423 was found to be involved in two π–cation bindings with the indole ring, whereas π–π stacked, π–π T-shaped and π–alkyl related bindings were noticed, connecting Ile235, Phe220, Ala546, Trp322 and Ile419. The fluorine atom was perceived interacting with Ile419.

### Docking studies of *h*-ENPP3 inhibitors

**Figure 5:**
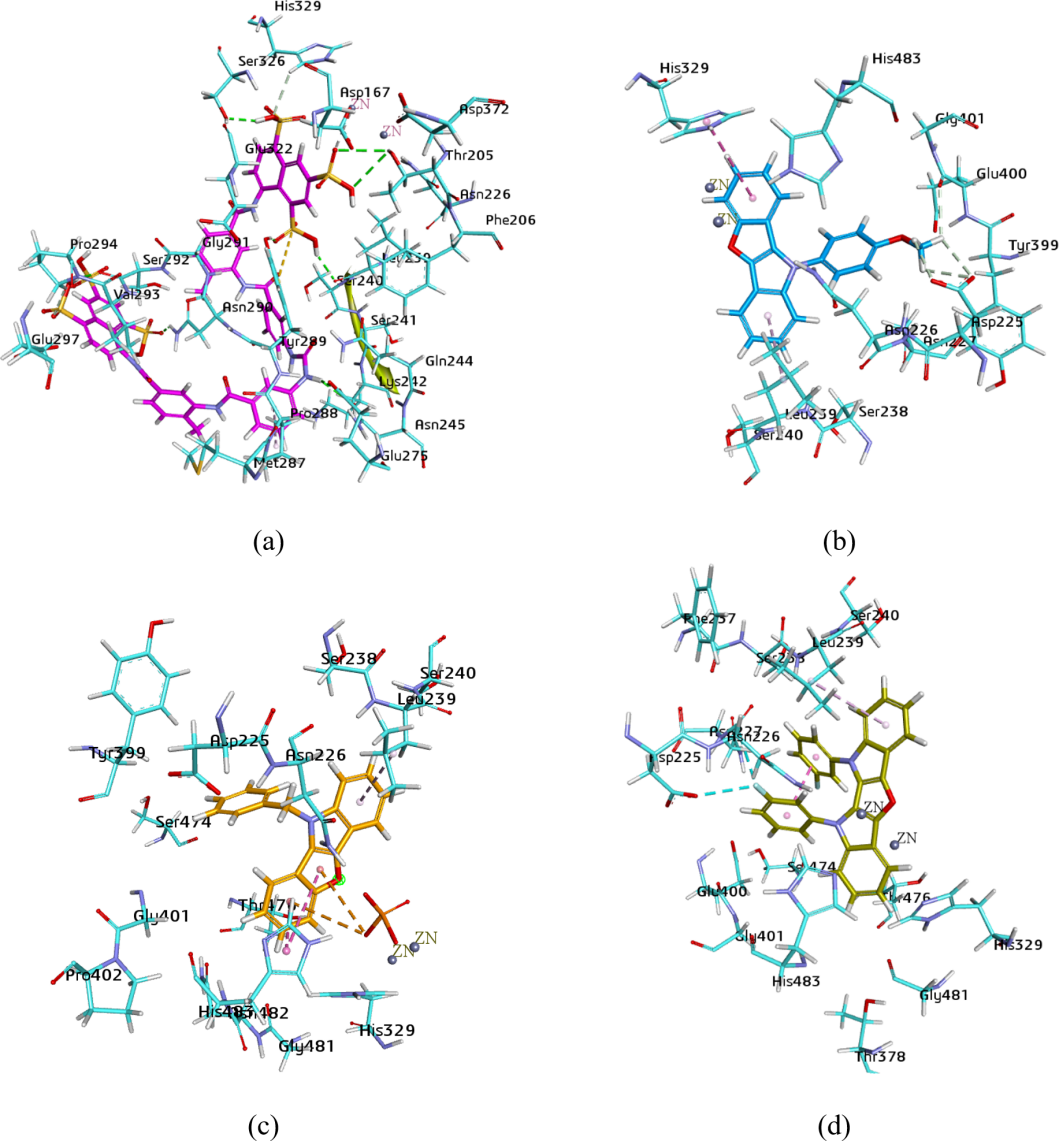
3D poses of docked selected inhibitors inside homology model of ENPP3. (a): suramin, (b): **5e**, (c): **5h**, (d): **6e**.

The binding of ENPP3 was studied for selective inhibitor **5e**. A dual affinity was observed for **5h** and **6e**. Suramin was used as a positive control. The 3D illustrations of these docked compounds are shown in Figure 5. The binding was observed in a large area of the modelled ENPP3 protein, inside and over the border of the active pocket, due to the bulky structure of suramin. The amino acids Asn290, Ser292, Asn245, Glu275, Tyr289, Leu239, Ser326, Thr205, His329 and Glu322 were involved in the hydrogen binding with suramin. One π–alkyl interaction was found with Pro288 and one metal interaction with zinc. Amino acid Tyr289 was connected with the sulfur atom as shown in Figure 5a. The hydrogen atoms of the methoxy group, located on the phenyl ring of compound **5e**, were engaged in carbon–hydrogen bonds with Asp225 and Glu400, as depicted in Figure 5b. However, phenyl rings of both indole and benzofuran scaffolds were inclined towards Leu239 and His329, respectively. When compound **5h** was taken into account, it was noticed that both five and six-membered rings of the benzofuran moiety were linked via π–π stacked relation with His483. However, the aromatic ring of indole demonstrated π–alkyl bonding by Leu239. The additional π–anion interaction was observed with the phosphate group and benzofuran. The 3D docking of compound **6e** revealed distinct bonding, due to the presence of a fluorine atom on the phenyl ring. The fluorine atom was attached to the side chains of Asn226 and Asp225. The aromatic ring of indole was coupled via π–alkyl line interactions with Leu239 (Figure 5d).

The docking studies of selected inhibitors on isozymes ENPP1 and ENPP3 modelled proteins were in accordance with in vitro experimental studies.

## Conclusion

In conclusion, we have reported a convenient strategy for the preparation of benzo[4,5]furo[3,2-*b*]indoles based on Suzuki–Miyaura coupling reactions followed by Pd-catalyzed double C,N-coupling. All products are potent inhibitors of nucleotide pyrophosphatases with sensitivities and selectivities several times greater than those of previously reported inhibitors of *h*-NPP-1 and *h*-NPP-3 [37]. Biological activities of these compounds are prone to changes of the substituents. The activities and selectivity of the compounds reported make them ideal targets for further applications in medicinal chemistry.

## Experimental

### General

All reagents were obtained from commercial sources and used without further purification. Reactions were conducted in oven-dried glassware under an inert atmosphere of argon. Concentrations (*c*) in the general procedures refer to the limiting reagent and are given in mmol/mL. Thin-layer chromatography (TLC) was carried out on Merck aluminium foil backed sheets precoated with 0.2 mm Kielselgel 60 F254. The spots were visualized by UV irradiation (λ = 254 nm). Column chromatography was performed on Fluka silica gel 60 (0.063–0.200 mm, 70–320 mesh) on a glass column. For the cation exchange column dowex 50WX8 H^+^ was used. Melting points (mp) were determined by the instrument Elektrothermal. ^1^H and ^13^C NMR spectra («Mercury-300 Varian» 300 MHz with Bruker AV 500 (75 MHz), Bruker AV 300 III (62.9 MHz), respectively) were recorded using TMS as an internal standard (0 ppm). NMR spectra are calibrated by solvent at 7.26 (CDCl_3_) and 77.23 (CDCl_3_) for ^13^C NMR spectra. ^13^C NMR spectra were measured proton decoupled. MS and HRMS spectra were recorded with a Finnigan MAT 95 XP spectrometer. IR spectra were recorded with a Nicolet 6700 FT-IR spectrometer.

**General procedure for the preparation of 3-bromo-2-(2-bromophenyl)benzofuran (3):** Analogously as previously described [31], 2,3-dibromobenzofuran (**1**), 2-bromophenylboronic acid (**2**), Pd(PPh_3_)_4_ (5 mol %) and K_3_PO_4_ were added to a Schlenk flask under Argon atmosphere. To the mixture 70 mL 1,4-dioxane and 10 mL distilled water were added. The reaction was heated at the desired temperature until the reaction was completed. The mixture was allowed to reach room temperature, diluted with water and extracted with dichloromethane. The organic layer was dried over Na_2_SO_4_, filtered and the solvent was evaporated in vacuo. The brown residue was purified by column chromatography (silica gel, heptane/ethyl acetate) to yield 3-bromo-2-(2-bromophenyl)benzofuran (**3**).

**General procedure for double C–N coupling with amine derivatives, exemplified by the synthesis of benzo[4,5]furo[3,2-*b*]indoles 5a–j.** Similarly as previously described [31], 1.1 equiv of amine **4** was added to a pressure tube charged with **3**, Pd_2_(dba)_3_ (5 mol %), BINAP (10 mol %) and 3 equiv of NaO*t-*Bu under Argon. The mixture was dissolved in anhydrous toluene (10 mL). The tube was sealed with a Teflon valve and stirred at 110 °C for 12 h. After the reaction was completed, the mixture was allowed to reach room temperature, worked up with water and extracted with dichloromethane. The combined organic layers were dried over sodium sulfate and concentrated under vacuum. The crude material was purified by flash column chromatography on silica gel (heptane/EtOAc) to yield benzo[4,5]furo[3,2-*b*]indoles **5a–j**.

### Nucleotide pyrophosphatase inhibition assay

The inhibition potential of all the sulphonate derivatives against NPP1 and NPP3, was determined by considering an already reported colorimetric method with minor modifications [38–41]. The reaction buffer was comprised of 50 mM Tris-hydrochloric acid, 5 mM magnesium chloride (MgCl_2_) and 0.1 mM zinc chloride (ZnCl_2_) with final pH 9.5. For initial screening of compounds, first enzyme and substrate parameters (concentration, temperature, time) were optimized. The reaction assay was performed at 100 µL well volume containing assay buffer, 10 µL of 1 mM test compound prepared in 10% DMSO, enzymes NPP1 (35 ng), NPP3 (94 ng) and assay buffer. The reaction mixture was incubated at 37 °C, 10 minutes for NPP1 and NPP3. Absorbance was taken at wavelength of 405 nm as pre-read by using a microplate reader (BioTek FLx800, Instruments, Inc. USA). After pre-read the artificial substrate *p*-nitrophenyl 5'-thymidine monophosphate (*p*NP-TMP), 400 µM for NPP1 and 600 µM in case of NPP3, was added followed by a second incubation at 37 °C, 15 minutes for NPP1 and NPP3. Absorbance was taken as after read. All the experiments were performed in triplicate. Compounds exhibiting more than 50% inhibition against either of NPP1 and NPP3 were further subjected to serial dilutions for the determination of IC_50_ values, using the nonlinear curve fitting program PRISM 5.0 (Graph Pad, San Diego, California, USA).

### Molecular docking studies

#### Homology modelling of human ENPP1 and ENPP3

Homology models of ENPP1 and ENPP3 were developed by our research group [34–36,42] because the X-ray crystallographic structures were not available in protein data bank (PDB). For the prediction of the 3D homology prototype, the reported structure of mouse ENPP1 with PDB ID 4B56, was used as standard pattern [43]. Molecular Operating Environment (2014. 0901) was used for this purpose. The modeled crystal structures manifested 80% for ENPP1 and 52% in case of ENPP3, amino acids sequence similarity with template, mouse ENPP1. The RMSD for ENPP1 was 0.613 Å and 1.349 Å for ENPP3, over 816 and 811 residues in comparison to the template, respectively. The Ramachandran plot represented promising stereochemical properties as above 98% amino acids were engaged in core and selected region of homology models [44].

#### Preparation of ligands and proteins

The structures of the most active compounds were drawn by utilizing the MOE builder tool [45]. After introducing hydrogen atoms and charges to the prepared structures, the MMFF94x force field was applied for energy minimization with root mean square deviation (RMSD) of 0.01 kcal/mol Å [46]. Modeled protein structures of ENPP1 and ENPP3 were protonated and the energy was minimized with the help of the AMBER99 force field and an RMSD gradient of 0.05 kcal/mol Å.

#### Docking analysis

Apparent binding interactions and binding energies of the selected compounds within the binding pocket of modeled proteins were found out by using MOE (2014.0901). MOE site finder was used to locate the binding site while default setting of the triangular matcher placement method in combination with GBVI/WSA ∆G, were used to find docking calculations and scoring functions. Out of 40 conformations for each compound, the conformations with lowest free binding energies and S score were selected for visualization by using Discovery studio visualizer DS [47–48].

### X-ray structure determination

An X-ray quality crystal of **5d** was selected in Fomblin YR-1800 perfluoroether (Alfa Aesar) at ambient temperature. The sample was cooled to 123(3) K during the measurement. The data was collected on a Bruker D8 QUEST diffractometer using Mo Kα radiation (λ = 0.71073 Å). The structure was solved by direct methods (SHELXTL) and refined by full-matrix least squares procedures (SHELXL-2014/7) [49–50]. Semi-empirical absorption corrections were applied (SADABS) [51]. All non-hydrogen atoms were refined anisotropically. Hydrogen atoms were included in the refinement at calculated positions using a riding model. Refinement details can be found in the cif file CCDC 1432173, which can be obtained free of charge from The Cambridge Crystallographic Data Centre via http://www.ccdc.cam.ac.uk/data_request/cif.

## Supporting Information

File 1 Additional experimental and analytical data, and NMR spectra of synthesized compounds.
