# Supplementary material for: Palladium-catalyzed synthesis and nucleotide pyrophosphatase inhibition of benzo[4,5]furo[3,2-b]indoles
Source: Beilstein J Org Chem. 2019 Nov 22;15:2830–9. doi: 10.3762/bjoc.15.276 (PMC6880817; doi:10.3762/bjoc.15.276)
Supplement: File 1 — Additional experimental and analytical data, and NMR spectra of synthesized compounds. [file Beilstein_J_Org_Chem-15-2830-s001.pdf]

BEILSTEIN JOURNAL OF ORGANIC CHEMISTRY

Supporting Information

for

Palladium-catalyzed synthesis and nucleotide pyrophosphatase inhibition of benzo[4,5]furo[3,2-*b*]indoles

Hoang Huy Do, Saif Ullah, Alexander Villinger, Joanna Lecka, Jean Sévigny, Peter Ehlers, Jamshed Iqbal and Peter Langer

Beilstein J. Org. Chem. **2019,** *15,* 2830–2839. doi:10.3762/bjoc.15.276

Additional experimental and analytical data, and NMR spectra of synthesized compounds

License and Terms: This is a supporting information file under the terms of the Creative Commons Attribution License (http://creativecommons.org/licenses/by/4.0). Please note that the reuse, redistribution and reproduction in particular requires that the authors and source are credited.
The license is subject to the *Beilstein Journal of Organic Chemistry* terms and conditions: (https://www.beilstein-journals.org/bjoc)

Table of Contents

General information S2

Crystal data of **5c**.. S3

Analytical data.. S5

NMR spectra.. S9

Biological protocols S20

References S21

General information

General information. All used chemicals are commercially available and used without further purification. All reactions were carried out in dried pressure tubes under an argon atmosphere. Analytical TLC on Merck silica gel 60 F254 plates was visualized by fluorescence quenching. Column chromatography was performed on Merck Geduran Si 60 (0.063–0.200 mm). ^{1}H and ^{13}C NMR spectra («Mercury-300 Varian» 300 MHz with Bruker AV 500 (75 MHz), Bruker AV 300 III (62.9 MHz) respectively) were recorded using TMS as an internal standard (0 ppm). NMR spectra are calibrated by solvent at 7.26 ($CDCl_3$) and 77.23 ($CDCl_3$) for ^{13}C NMR Spectra. ^{13}C NMR spectra were measured proton decoupled. Multiplicities: s = singlet, d = doublet, dd = doublet of doublets, t = triplet, td = triplet of doublets, tt = triplet of triplets, m = multiplet, q = quartet. Nicolet 550 FT–IR spectrometer was used with ATR sampling technique for solids. Signal characterization: vw = very weak, w = weak, m = medium, s = strong, vs = very strong. Gas chromatography–mass spectrometry was performed on an Agilent HP-5890 instrument with an Agilent HP-5973 Mass Selective Detector (EI) and HP-5 capillary column using helium carrier gas. Agilent 1969A TOF mass spectrometer was used for ESI HRMS measurements. High-resolution MS (HRMS) was performed on a Finnigan MAT 95 XP. Single crystal X-Ray structure determination was carried out on a Bruker X8Apex diffractometer with CCD camera (Mo Ka radiation and graphite monochromator, a = 0.071073 Å). Melting points were determined on a Micro-Hot-Stage GalenTM III Cambridge Instruments. The melting points are not corrected.

Crystal Data of 5c

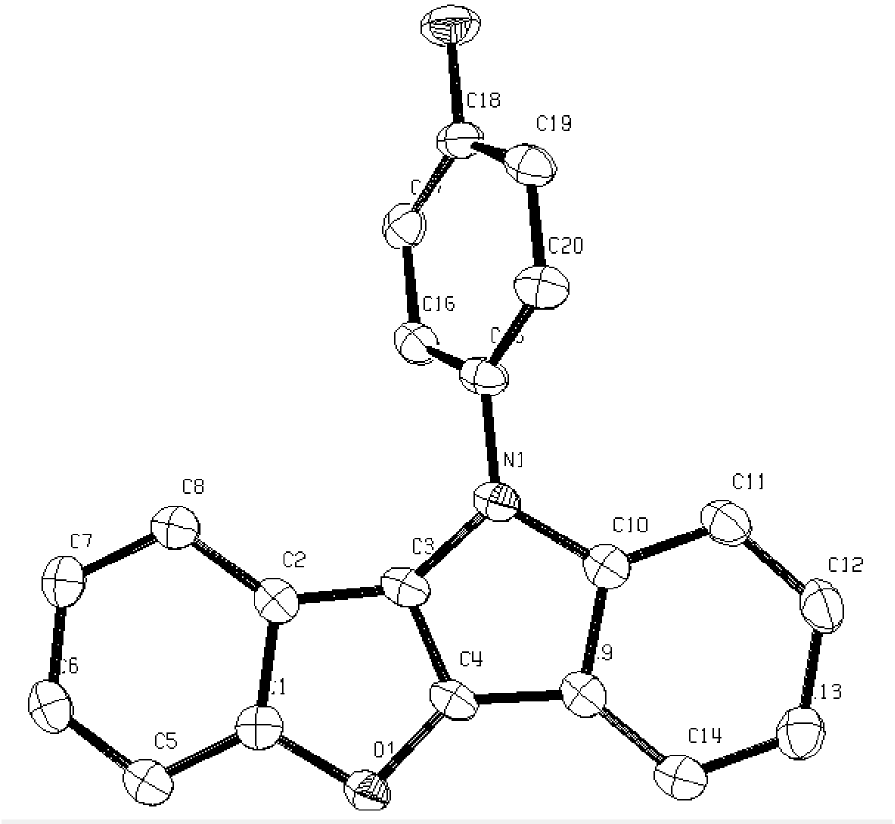

Figure 1. Chrystal structure of compound **5c**.

Chemical formula moiety	$C_{20} H_{12}FNO$
Chemical formula weight	301.31
Symmetry cell setting	monoclinic
Symmetry space group name H-M	'C 2/c'
Symmetry space group name Hall	'-C 2yc'
Symmetry Int Tables number	15
Cell length a	16.4374(11)
Cell length b	7.0067(5)
Cell length c	24.6717(16)
Cell angle alpha	90.00
Cell angle beta	102.856(3)
Cell angle gamma	90.00
Cell volume	2770.3(3)
Cell formula units Z	8
Exptl crystal description	block
Exptl crystal colour	colourless
Exptl crystal size max	0.45
Exptl crystal size mid	0.13
Exptl crystal size min	0.07
Exptl crystal density diffrn	1.445
Exptl crystal F 000	1248

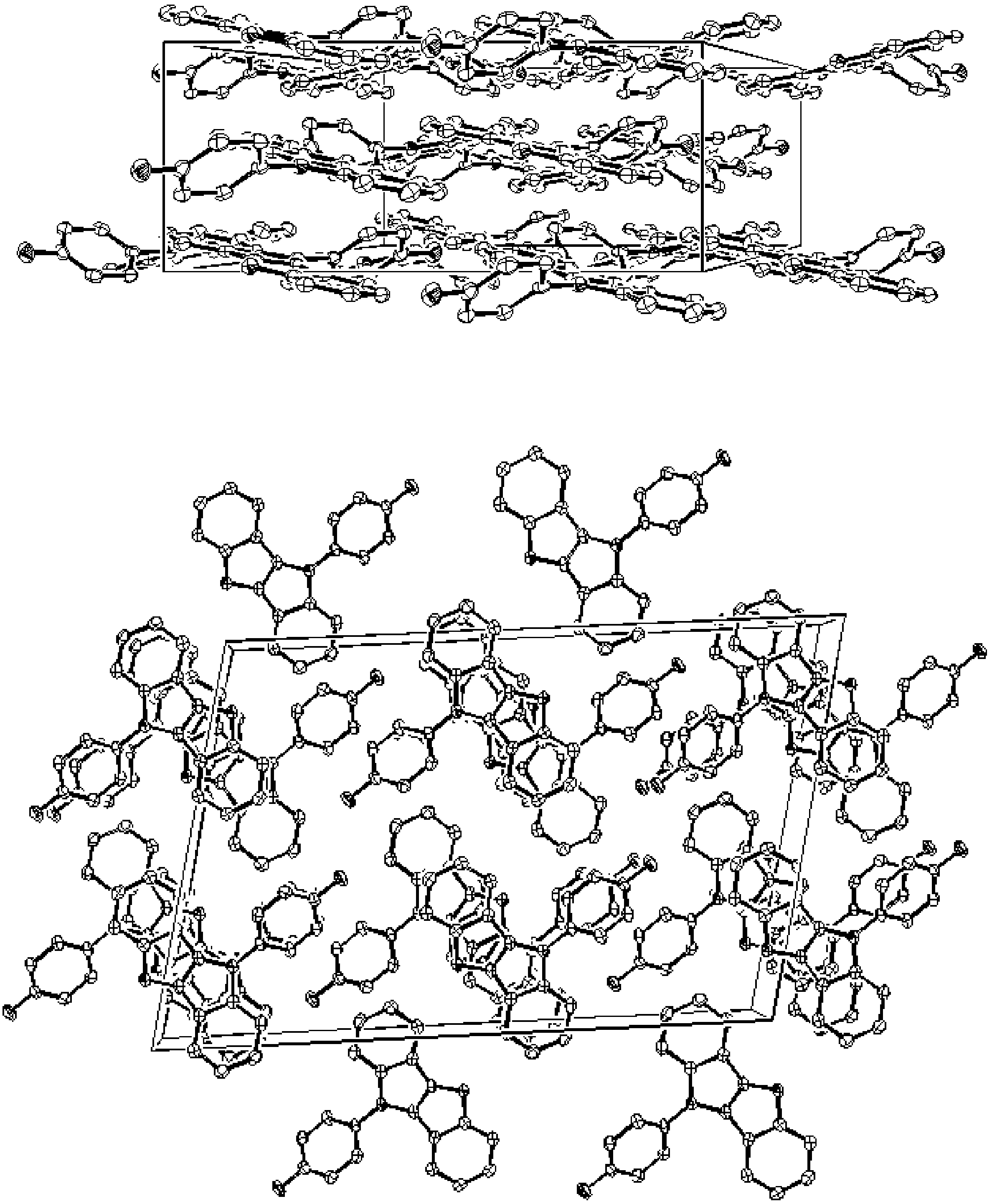

Figure 2. Chrystal lattice of compound **5c**

Analytical data

3-Bromo-2-(2-bromophenyl)benzofuran (3)

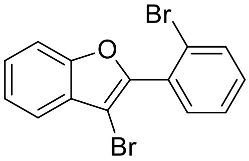

White solid, 84 %. ^{1}H NMR (250 MHz, $CDCl_3$) δ= 7.75 (d, ^{3}J = 7.9 Hz, 1H, CH_{Ar}), 7.65 – 7.50 (m, 3H, CH_{Ar}), 7.49 – 7.31 (m, 4H, CH_{Ar}). ^{13}C NMR (63 MHz, $CDCl_3$) δ= 154.0, 151.4 ($C_{Ar/Hetar}$), 133.6, 132.8, 131.5 (CH_{Ar}), 130.7, 128.5 ($C_{Ar/Hetar}$), 127.3, 125.9 (CH_{Ar}), 124.0 ($C_{Ar/Hetar}$), 123.7, 120.2, 111.8 (CH_{Ar}), 97.4 ($C_{Ar/Hetar}$). IR (ATR, cm^{-1}), ṽ = 3055 (w), 2953 (w), 1610 (w), 1591 (w), 1574 (w), 1562 (w), 1477 (w), 1460 (w), 1444 (s), 1344 (w). MS (EI, 70 eV), m/z (%) = 353 (100), 243 (32), 192 (12), 163 (57), 137 (9), 122 (8), 82 (11). HRMS (EI, 70 eV), [$C_{14}H_8OBr_2$] = 349.89364, found 349.89317, [$C_{14}H_8OBr^{81}Br$] = 351.89160, found 351.89126, [$C_{14}H_8O^{81}Br_2$] = 353.88955, found 353.88937.

10-Phenyl-10*H*-benzofuro[3,2-*b*]indole (5a)

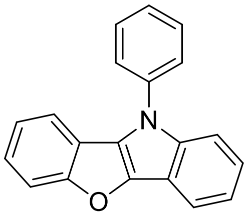

White solid, 63 %. M.p., 137 – 139 °C. ^{1}H NMR (250 MHz, $CDCl_3$) δ 7.95 – 7.87 (m, 1H, CH_{Ar}), 7.75 – 7.58 (m, 6H, CH_{Ar}), 7.55 – 7.42 (m, 2H, CH_{Ar}), 7.38 – 7.28 (m, 3H, CH_{Ar}), 7.25 – 7.20 (m, 1H, CH_{Ar}). ^{13}C NMR (63 MHz, $CDCl_3$) δ 159.3, 143.7, 139.6, 138.4 (C_{Ar}), 129.8 ($2CH_{Ph}$), 126.8 (CH_{Ar}), 126.5 (C_{Ar}), 125.08 ($2CH_{Ph}$), 124.0, 123.2, 122.5, 120.7 (CH_{Ar}), 118.6(C_{Ar}), 118.4, 117.4 (CH_{Ar}), 114.6 (C_{Ar}), 112.7, 111.3 (CH_{Ar}). IR (ATR, cm^{-1}), ṽ = 3063 (w), 3045 (w), 2922 (w), 2850 (w), 1595 (w), 1547 (w), 1508 (m), 1498 (m), 1487 (m), 1450 (s), 1435 (m), 1417 (w). MS (EI, 70 eV), m/z (%) = 283 (100), 254 (44), 226 (4), 206 (5), 177 (7), 151 (8), 126 (3). HRMS (EI, 70 eV), m/z (%) [$C_{20}H_{13}ON$], 283.09917, found 283.09843.

10-(*p*-tolyl)-10*H*-benzofuro[3,2-*b*]indole (5b)

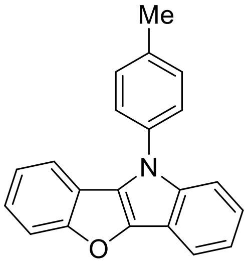

White solid, 75 %.M.p., 134-135 °C. ^{1}H NMR (300 MHz, $CDCl_3$) δ 7.93 – 7.86 (m, 1H, CH_{Ar}), 7.68 – 7.49 (m, 5H, CH_{Ar}), 7.45 – 7.40 (m, 2H, CH_{Ar}), 7.37 – 7.26 (m, 3H, CH_{Ar}), 7.25 – 7.20 (m, 1H, CH_{Ar}), 2.51 (s, 3H, CH_3). ^{13}C NMR (63 MHz, $CDCl_3$) δ 159.5, 143.6, 139.8, 136.8, 135.9 (C_{Ar}), 130.5 ($2CH_{Ar}$), 126.8 (C_{Ar}), 125.1 ($2CH_{Ar}$), 124.0, 123.2, 122.6, 120.7 (CH_{Ar}), 118.8(C_{Ar}), 118.6, 117.5 (CH_{Ar}), 114.6 (C_{Ar}), 112.8, 111.4 (CH_{Ar}), 21.3 (CH_3). IR (ATR, cm^{-1}), ṽ = 3051 (w), 3034 (w), 2918 (w), 1614 (w), 1549 (w), 1520 (s), 1485 (w), 1454 (s), 1441 (m). MS (EI, 70 eV), m/z (%) =, 297 (100), 282 (31), 268 (12), 254 (22), 226 (2), 206 (4), 190 (2), 177 (5), 165 (4), 149 (7). HRMS (EI, 70 eV) [$C_{21}H_{15}ON$] = 297.11482, found 297.11421.

10-(4-Fluorophenyl)-10*H*-benzofuro[3,2-*b*]indole (5c)

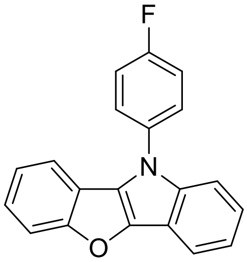

White solid, 79 %.M.p., 167 – 168 °C. ^{1}H NMR (300 MHz, $CDCl_3$) δ 7.94 – 7.84 (m, 1H, CH_{Ar}), 7.68 – 7.60 (m, 3H, CH_{Ar}), 7.59 – 7.52 (m, 1H, CH_{Ar}), 7.47 – 7.40 (m, 1H, CH_{Ar}), 7.37 – 7.27 (m, 5H, CH_{Ar}), 7.26 – 7.20 (m, 1H, CH_{Ar}). ^{19}F NMR (282 MHz, $CDCl_3$) δ -114.58. ^{13}C NMR (63 MHz, $CDCl_3$) δ 163.2 (d, ^{1}J = 248.3 Hz, CF), 159.3, 143.6, 139.8 (C_{Ar}), 134.4 (d, ^{4}J = 3.0 Hz, C_{Ar}), 126.9 (d, ^{3}J = 8.5 Hz, $2CH_{Ar}$), 126.6 (C_{Ar}),124.1, 123.3, 122.6, 120.8 (CH_{Ar}), 118.5 (C_{Ar}), 118.1, 117.5 (CH_{Ar}), 116.7 (d, ^{2}J = 22.7 Hz, $2CH_{Ar}$), 114.5 (C_{Ar}), 112.8, 111.0(CH_{Ar}). IR (ATR, cm^{-1}), ṽ = 3063 (m), 1911 (w), 1874 (w), 1837 (w), 1549 (w), 1513 (s), 1498 (s), 1453 (s), 1441 (m), 1413 (w). MS (EI, 70 eV), m/z (%) =, 301 (100), 272 (45), 251 (7), 224 (2), 196 (3), 177 (4), 150 (11). HRMS (EI, 70 eV) [$C_{20}H_{12}ONF$] = 301.08974, found 301.08924.

10-(3-(Trifluoromethyl)phenyl)-10*H*-benzofuro[3,2-*b*]indole (5d)

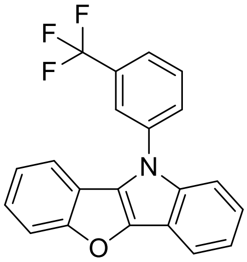

White solid, 81 %. M.p., 178 - 179 °C. ^{1}H NMR (300 MHz, $CDCl_3$) δ 7.96 (s, 1H, CH_{Ar}), 7.92 – 7.84 (m, 2H, CH_{Ar}), 7.79 – 7.57 (m, 4H, CH_{Ar}), 7.47 – 7.40 (m, 1H, CH_{Ar}), 7.35 – 7.27 (m, 3H, CH_{Ar}), 7.25 – 7.20 (m, 1H, CH_{Ar}). ^{19}F NMR (282 MHz, $CDCl_3$) δ -62.67. ^{13}C NMR (75 MHz, $CDCl_3$) δ 159.3, 144.2, 139.4, 139.1 (C_{Ar}), 132.5 (q, ^{2}J = 32.9 Hz, *C*CF_3), 130.5 (CH_{Ar}), 128.0 (q, ^{4}J = 1.0 Hz, CH_{Ar}), 126.1 (C_{Ar}), 124.3, 124.0 (CH_{Ar}), 123.7 (q, ^{1}J = 274.4 Hz, CF_3), 123.3 (q, ^{3}J = 3.8 Hz, CH_{Ar}), 122.8 (CH_{Ar}), 121.8 (q, ^{3}J = 3.8 Hz, CH_{Ar}), 121.4 (CH_{Ar}), 118.4 (C_{Ar}), 118.1, 117.7(CH_{Ar}), 115.1 (C_{Ar}), 112.9, 110.9 (CH_{Ar}). IR (ATR, cm^{-1}), ṽ = 3061 (w), 1612 (w), 1593 (w), 1576 (w), 1512 (m), 1495 (m), 1483 (w), 1456 (S), 1439 (m), 1417 (w). MS (EI, 70 eV), m/z (%) =, 351 (100), 332 (4), 322 (18), 302 (3), 282 (13), 254 (27), 226 (3), 206 (3), 175 (9), 151 (6). HRMS (EI, 70 eV) [$C_{21}H_{12}ONF_3$] = 351.08655, found 351.08586.

10-(4-Methoxyphenyl)-10*H*-benzofuro[3,2-*b*]indole (5e)

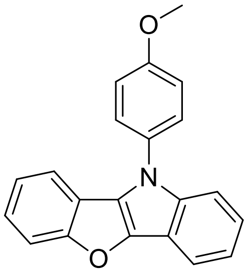

White solid, 65 %. M.p., 139 - 141 °C. ^{1}H NMR (300 MHz, $CDCl_3$) δ 7.91 – 7.82 (m, 1H, CH_{Ar}), 7.61 (d, ^{3}J = 8.2 Hz, 1H, CH_{Ar}), 7.58 – 7.49 (m, 3H, CH_{Ar}), 7.46 – 7.40 (m, 1H, CH_{Ar}), 7.33 – 7.27 (m, 1H, CH_{Ar}), 7.26 – 7.16 (m, 3H, CH_{Ar}), 7.13 – 7.06 (m, 2H, CH_{Ar}), 3.89 (s, 3H, OCH_3). ^{13}C NMR (75 MHz, $CDCl_3$) δ 159.42, 158.62, 143.37, 140.12, 131.30, 127.03 (C_{Ar}), 126.7 ($2CH_{Ar}$), 124.0, 123.1, 122.6, 120.6 (CH_{Ar}), 118.8 (C_{Ar}), 118.4, 117.4 (CH_{Ar}), 115.1 ($2CH_{Ar}$), 114.4 (C_{Ar}), 112.8, 111.3 (CH_{Ar}), 55.7

(CH_3). IR (ATR, cm^{-1}), ṽ = 3005 (m), 2951 (m), 2924 (m), 2872 (m), 2850 (m), 2831 (m), 1514 (s), 1504 (s), 1452 (s), 1435 (m). MS (EI, 70 eV), m/z (%) =, 313 (100), 298 (13), 282 (8), 270 (21), 254 (10), 241 (14), 157 (7), 121 (7). HRMS (EI, 70 eV) [$C_{21}H_{15}O_2N$] = 313.10973, found 313.10934.

10-(3,4-Dimethoxyphenyl)-10*H*-benzofuro[3,2-*b*]indole (5f)

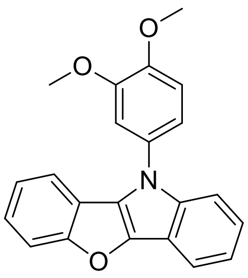

White solid, 51 %. M.p., 175 - 176 °C. ^1H NMR (300 MHz, $CDCl_3$) δ 7.85 – 7.77 (m, 1H, CH_{Ar}), 7.61 – 7.48 (m, 2H, CH_{Ar}), 7.45 – 7.39 (m, 1H, CH_{Ar}), 7.28 – 7.18 (m, 3H, CH_{Ar}), 7.17 – 7.12 (m, 2H, CH_{Ar}), 7.09 (d, J = 2.4 Hz, 1H, CH_{Ar}), 7.00 (d, ^3J = 8.5 Hz, 1H, CH_{Ar}), 3.93 (s, 3H, OCH_3), 3.85 (s, 3H, OCH_3). ^{13}C NMR (75 MHz, $CDCl_3$) δ 159.5, 149.9, 148.2, 143.4, 140.1, 131.5, 127.01 (C_{Ar}), 124.1, 123.2, 122.7, 120.6 (CH_{Ar}), 118.8 (C_{Ar}), 118.3, 117.7, 117.5 (CH_{Ar}), 114.4 (C_{Ar}), 112.9, 111.8, 111.4, 109.4 (CH_{Ar}), 56.4, 56.3 (OCH_3). IR (ATR, cm^{-1}), ṽ = 3063 (w), 3003 (w), 2951 (w), 2928 (w), 2850 (w), 2833 (w), 1595 (m), 1574 (w), 1547 (w), 1514 (s), 1450 (s), 1439 (s), 1417 (m). MS (EI, 70 eV), m/z (%) = 343 (100), 328 (14), 312 (10), 256 (14), 228 (8), 206 (9), 177 (6), 151 (6), 120 (4). HRMS (EI, 70 eV), [$C_{22}H_{17}O_3N$] = 343.12029, found 343.12067.

10-(4-(*tert*-Butyl)phenyl)-10*H*-benzofuro[3,2-*b*]indole (5g)

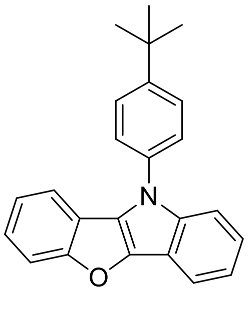

White solid, 84 %. M.p., 195 - 197 °C. ^1H NMR (300 MHz, $CDCl_3$) δ 7.94 – 7.86 (m, 1H, CH_{Ar}), 7.72 – 7.59 (m, 6H, CH_{Ar}), 7.58 – 7.53 (m, 1H, CH_{Ar}), 7.37 – 7.26 (m, 3H, CH_{Ar}), 7.26 – 7.21 (m, 1H, CH_{Ar}), 1.45 (s, 9H, $3CH_3$). ^{13}C NMR (63 MHz, $CDCl_3$) δ 159.5, 150.0, 143.7, 139.8, 135.8 (C_{Ar}), 126.7, 124.7 ($2CH_{Ar}$), 124.0, 123.2, 122.6, 120.7 (CH_{Ar}), 118.9 (C_{Ar}), 118.7, 117.5 (CH_{Ar}), 114.6 (C_{Ar}), 112.8, 111.6 (CH_{Ar}), 34.9 (*C*(CH_3)$_3$), 31.6 ($3CH_3$) (one signal of C could not be detected). IR (ATR, cm^{-1}), ṽ = 3059 (w), 2958 (m), 2901 (w), 2864 (w), 1605 (w), 1547 (w), 1520 (m), 1504 (m), 1479 (w), 1454 (s), 1439 (m). MS (EI, 70 eV), m/z (%) =, 339 (100), 324 (36), 309 (8), 282 (20), 254 (10), 206 (8), 177 (3), 162 (4), 148 (14). HRMS (EI, 70 eV) [$C_{24}H_{21}ON$] = 339.16177, found 339.16142.

10-Benzyl-10*H*-benzofuro[3,2-*b*]indole (5h)

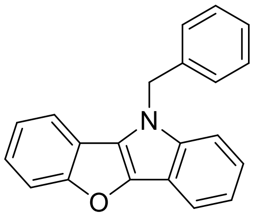

White solid, 67 %. M.p., 136 - 137 °C. ^1H NMR (250 MHz, $CDCl_3$) δ 7.89 – 7.81 (m, 1H, CH_{Ar}), 7.62 – 7.54 (m, 1H, CH_{Ar}), 7.44 – 7.32 (m, 2H, CH_{Ar}), 7.30 – 7.26 (m, 1H, CH_{Ar}), 7.25 – 7.13 (m, 8H, CH_{Ar}), 5.56

(s, 2H, CH_2). ^{13}C NMR (63 MHz, $CDCl_3$) δ 159.3, 142.7, 140.2, 137.5 (C_{Ar}), 129.1 ($2CH_{Ar}$), 127.9 (CH_{Ar}), 127.3 (C_{Ar}), 126.8 ($2CH_{Ar}$), 123.9, 122.7, 122.7, 119.9 (CH_{Ar}), 118.9 (C_{Ar}), 117.9, 117.5 (CH_{Ar}), 114.0 (C_{Ar}), 112.8, 110.5 (CH_{Ar}), 49.0 (CH_2). IR (ATR, cm^{-1}), ṽ = 3057 (w), 1516 (w), 1495 (w), 1456 (s), 1441 (m), 1417 (w), 1394 (m), 1354 (m). MS (EI, 70 eV), m/z (%) = 297 (100), 268 (4), 220 (8), 206 (81), 190 (1), 177 (19), 151 (21). HRMS (EI, 70 eV), [$C_{21}H_{15}ON$] = 297.11482, found 297.11421.

<u>10-Heptyl-10*H*-benzofuro[3,2-*b*]indole (5i)</u>

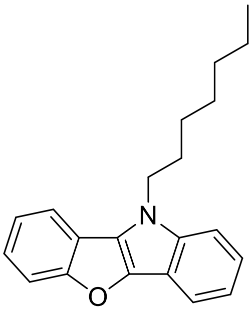

White solid, 53 %. M.p., 54 - 55 °C. ^1H NMR (300 MHz, $CDCl_3$) δ 7.85 (d, ^3J = 7.8 Hz, 1H, CH_{Ar}), 7.78 – 7.71 (m, 1H, CH_{Ar}), 7.68 – 7.60 (m, 1H, CH_{Ar}), 7.46 (d, ^3J = 8.3 Hz, 1H, CH_{Ar}), 7.38 – 7.27 (m, 3H, CH_{Ar}), 7.25 – 7.18 (m, 1H, CH_{Ar}), 4.43 (t, ^3J = 7.1 Hz, 2H, CH_2), 2.08 – 1.85 (m, 2H, CH_2), 1.47 – 1.14 (m, 8H, $4CH_2$), 0.85 (t, ^3J = 6.8 Hz, 3H, CH_3). ^{13}C NMR (63 MHz, $CDCl_3$) δ 159.4, 142.4, 139.7, 127.0 (C_{Ar}), 123.8, 122.7, 122.3, 119.4 (CH_{Ar}), 119.0 (C_{Ar}), 117.8, 117.4 (CH_{Ar}), 113.6 (C_{Ar}), 112.9, 110.3 (CH_{Ar}), 45.6, 31.8, 30.7, 29.2, 27.2, 22.7 (CH_2), 14.2 (CH_3). IR (ATR, cm^{-1}), ṽ = 3064 (w), 3053 (w), 2949 (m), 2937 (m), 2918 (m), 2868 (w), 2850 (m), 1543 (w), 1514 (w), 1456 (s), 1441 (m). MS (EI, 70 eV), m/z (%) =, 305 (100), 276 (2), 220 (86), 206 (10), 190 (5), 165 (12), 151 (7). HRMS (EI, 70 eV), [$C_{21}H_{23}ON$] = 305.17741, found 305.17746.

<u>10-Cyclohexyl-10*H*-benzofuro[3,2-*b*]indole (5j)</u>

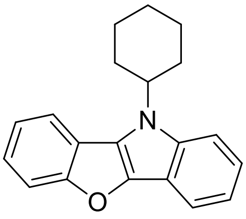

White solid, 57 % .M.p., 163 - 164 °C. ^1H NMR (300 MHz, $CDCl_3$) δ 7.90 – 7.75 (m, 2H, $2CH_{Ar}$), 7.63 – 7.55 (m, 1H, CH_{Ar}), 7.49 (d, ^3J = 8.5 Hz, 1H, CH_{Ar}), 7.34 – 7.27 (m, 2H, CH_{Ar}), 7.24 – 7.10 (m, 2H, CH_{Ar}), 4.56 – 4.37 (m, 1H), 2.21 – 1.82 (m, 7H), 1.65 – 1.40 (m, 3H). ^{13}C NMR (63 MHz, $CDCl_3$) δ 159.4, 143.3, 139.2, 125.3 (C_{Ar}), 123.7, 122.6, 122.4, 119.7 (CH_{Ar}), 119.3 (C_{Ar}), 119.2, 117.4, 112.9, 110.5 (CH_{Ar}), 54.8 (CH), 33.0, 26.2 ($2CH_2$), 25.7 (CH_2) (one signal could not be detected). IR (ATR, cm^{-1}), ṽ = 3064 (w), 3053 (w), 2949 (m), 2937 (m), 2918 (m), 2868 (w), 2850 (m), 1543 (w), 1514 (w), 1456 (s), 1441 (m). 3064 (w), 2935 (m), 2920 (m), 2852 (m), 1504 (w), 1454 (s), 1417 (w), MS (EI, 70 eV), m/z (%) =, 289 (92), 207 (100), 177 (13), 151 (15). HRMS (EI, 70 eV) [$C_{20}H_{19}ON$] = 289.14612, found 289.14568.

NMR spectra

3-Bromo-2-(2-bromophenyl)benzofuran (3)

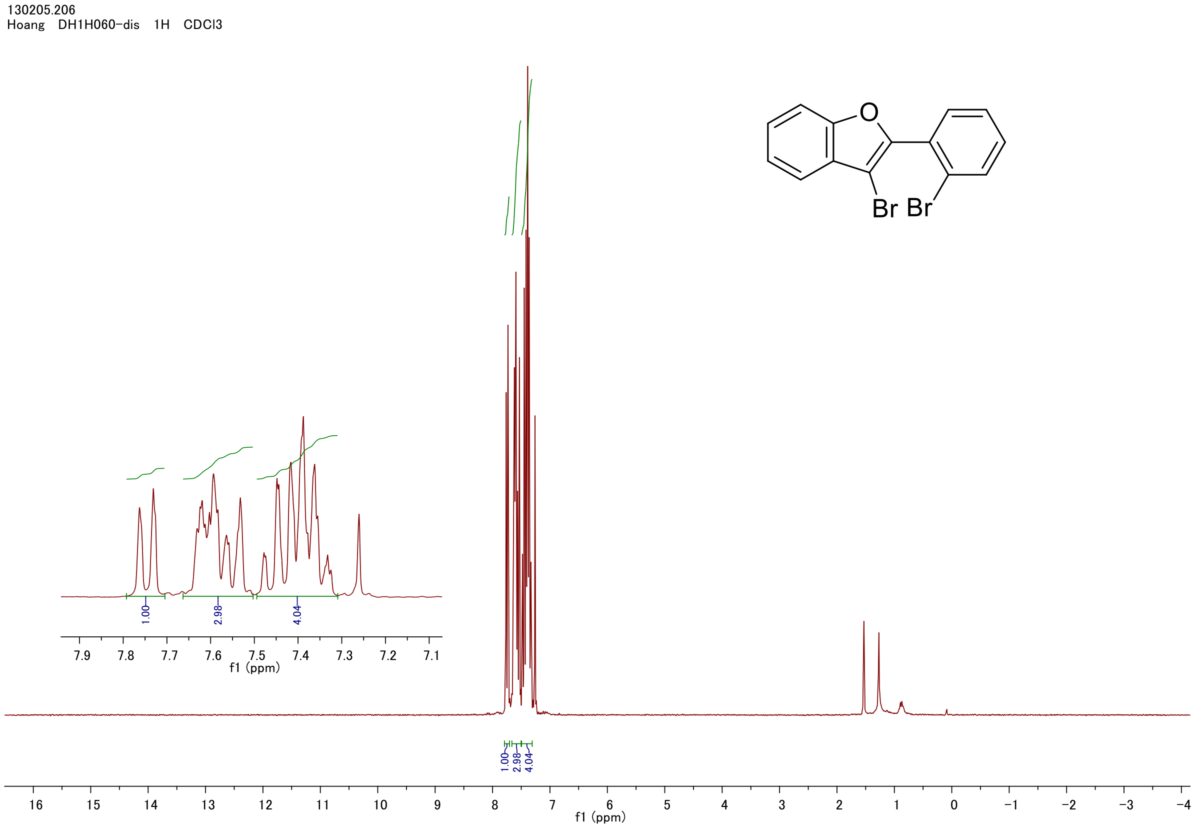

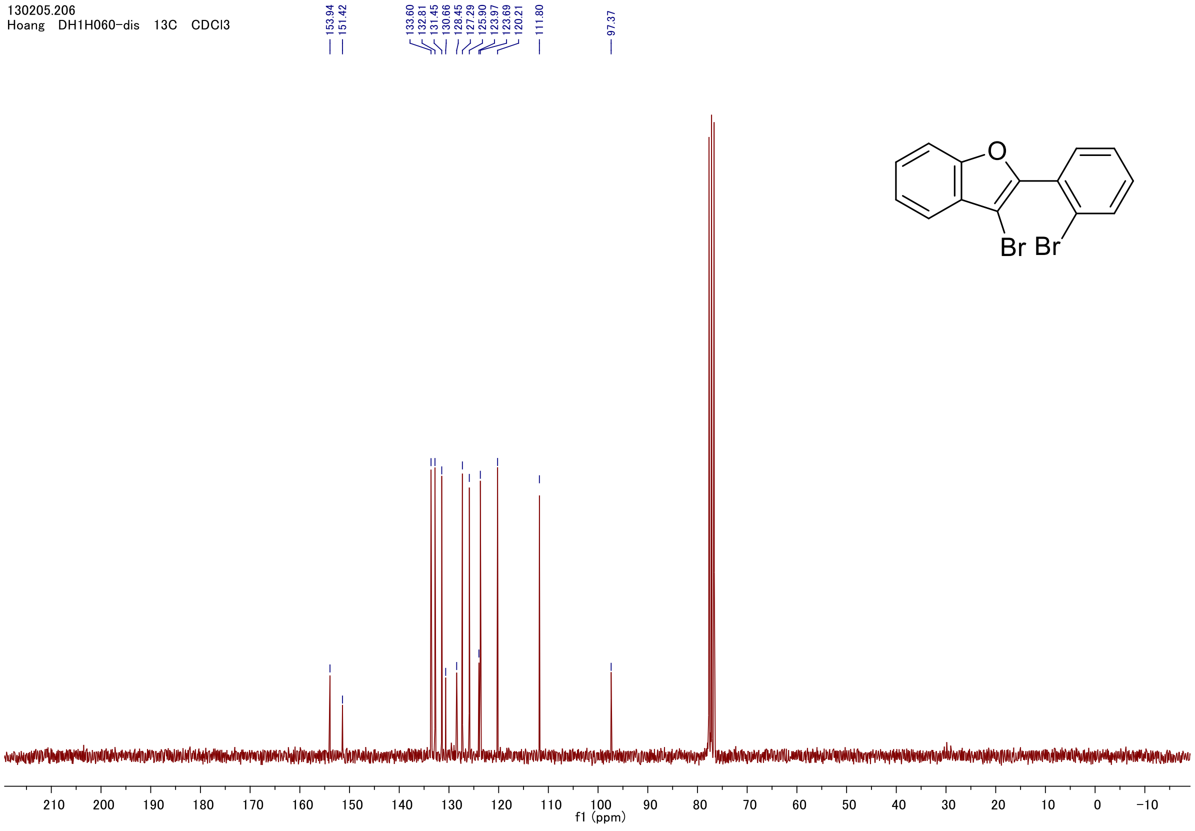

10-Phenyl-10*H*-benzofuro[3,2-*b*]indole (5a)

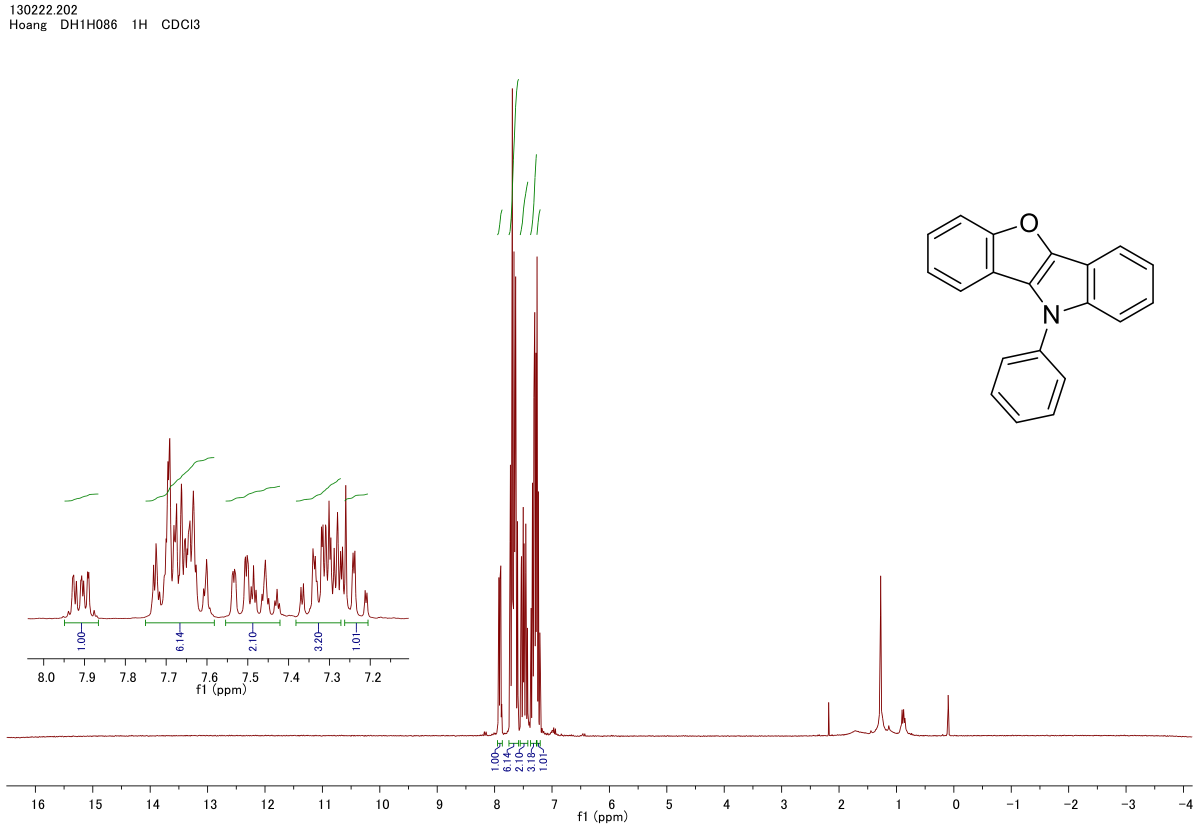

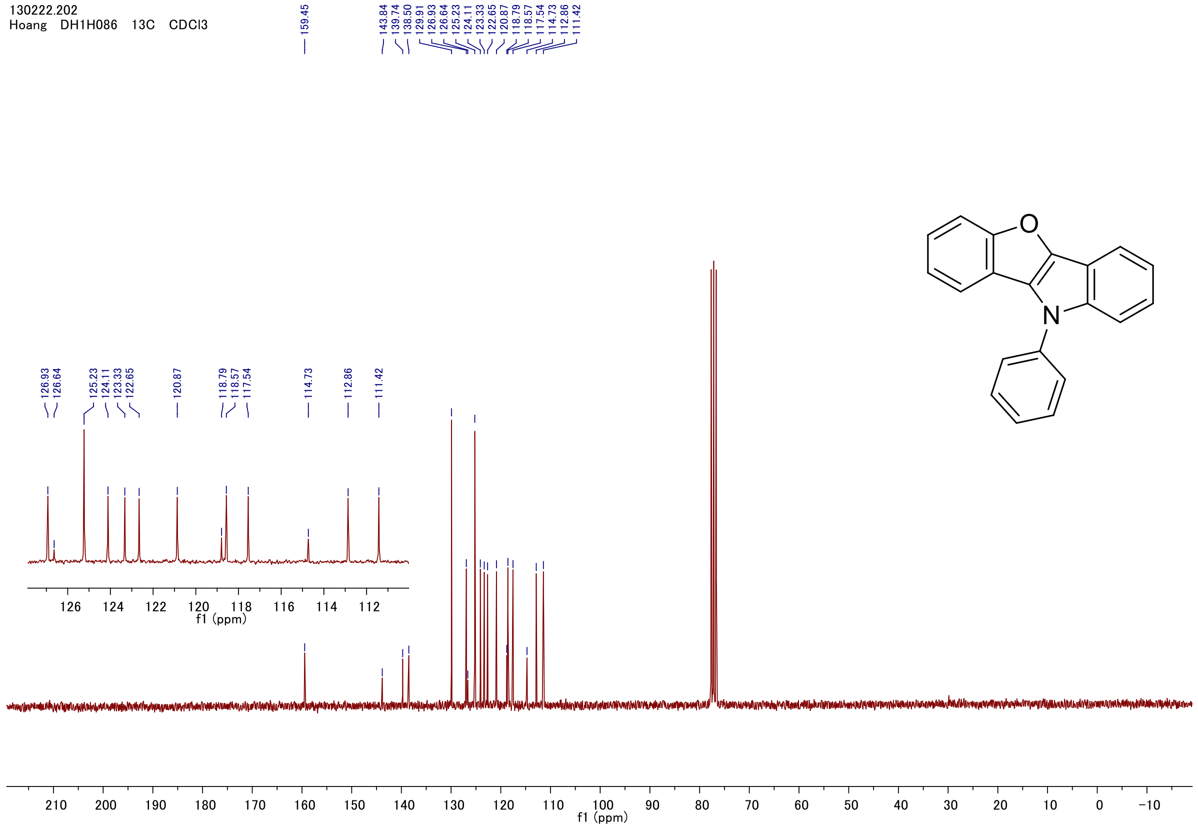

10-(*p*-Tolyl)-10*H*-benzofuro[3,2-*b*]indole (5b)

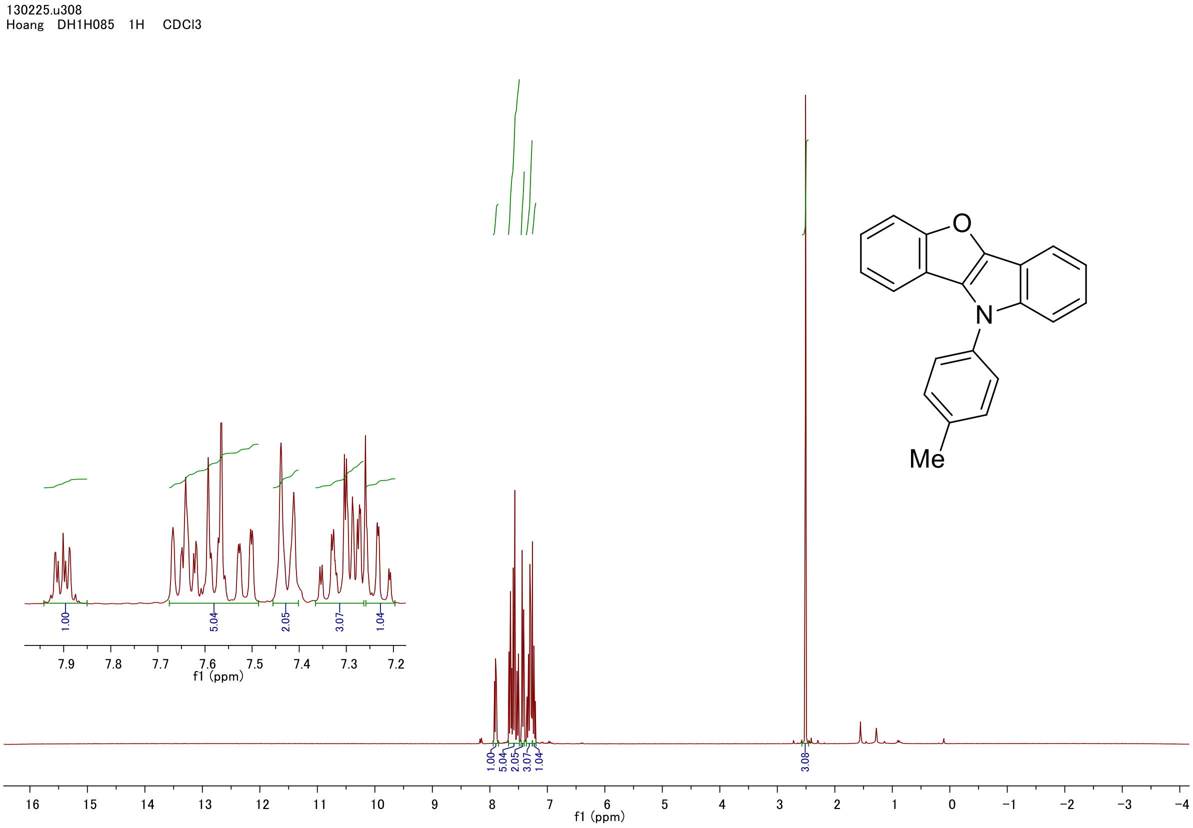

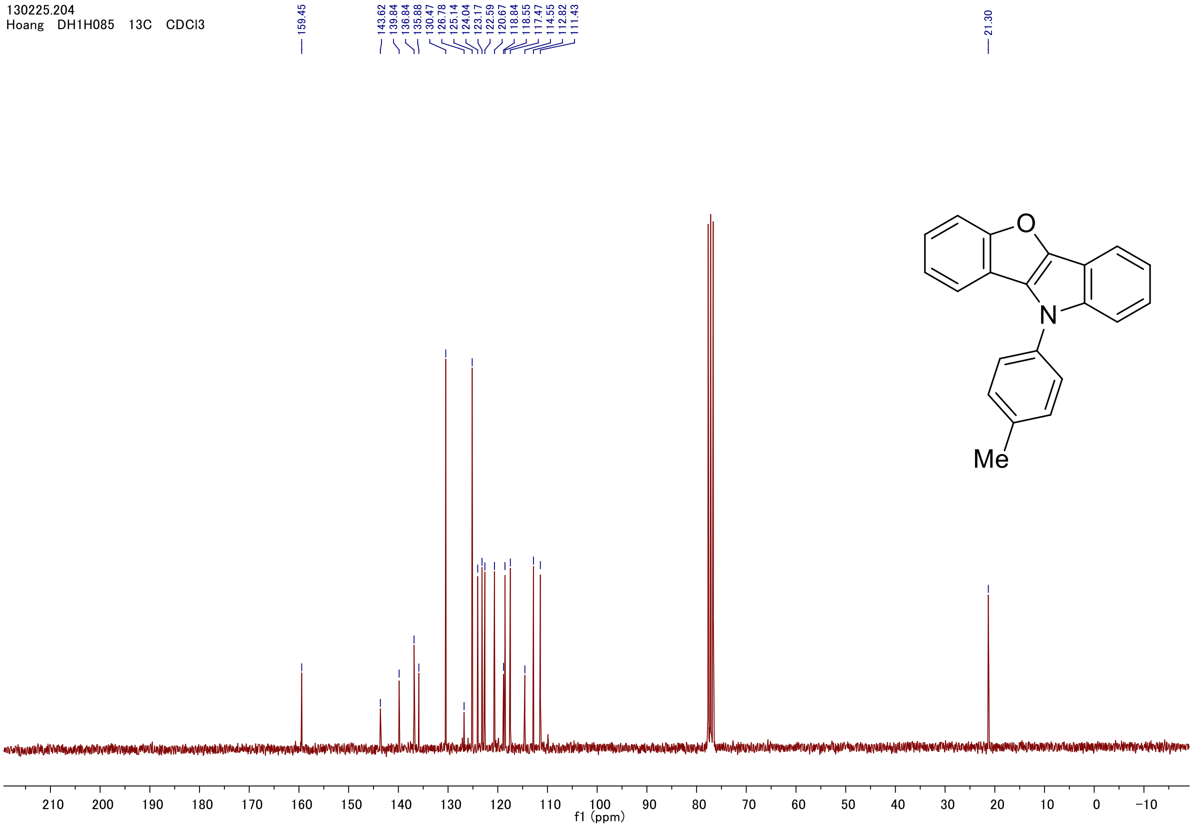

10-(4-Fluorophenyl)-10*H*-benzofuro[3,2-*b*]indole (5c)

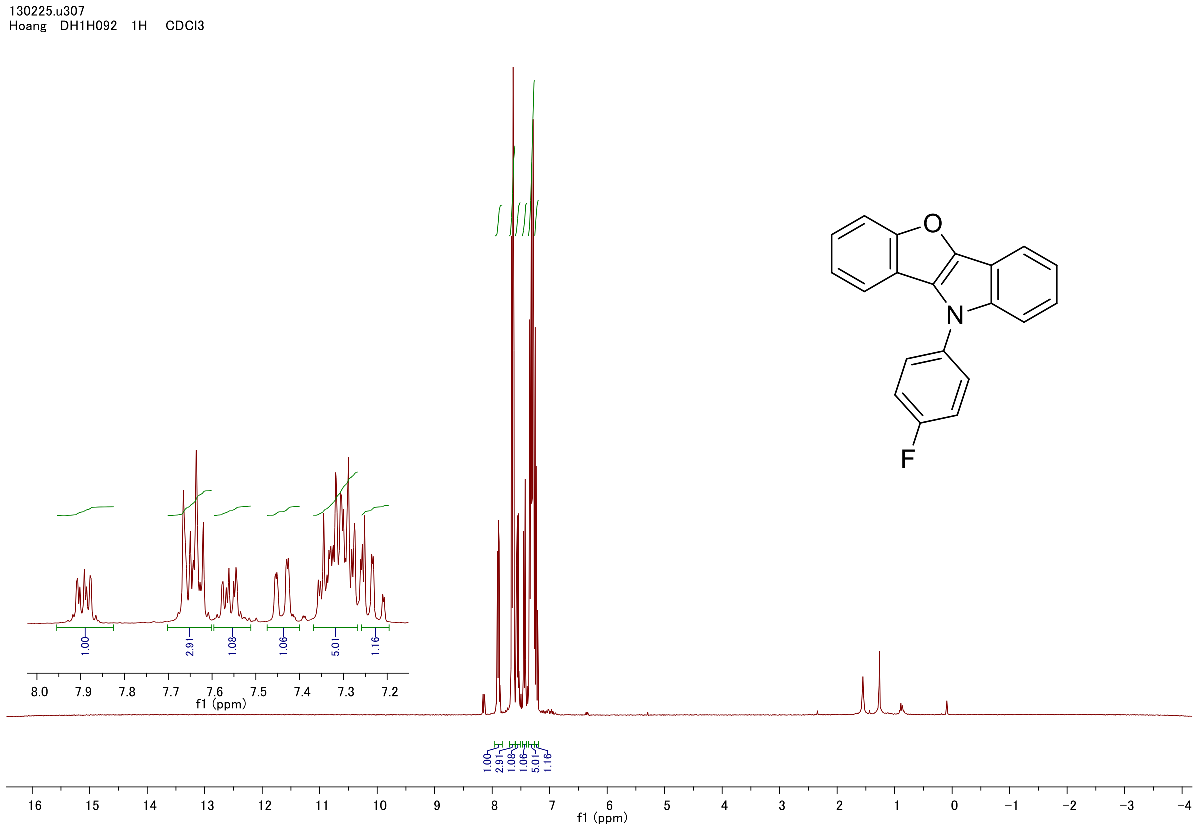

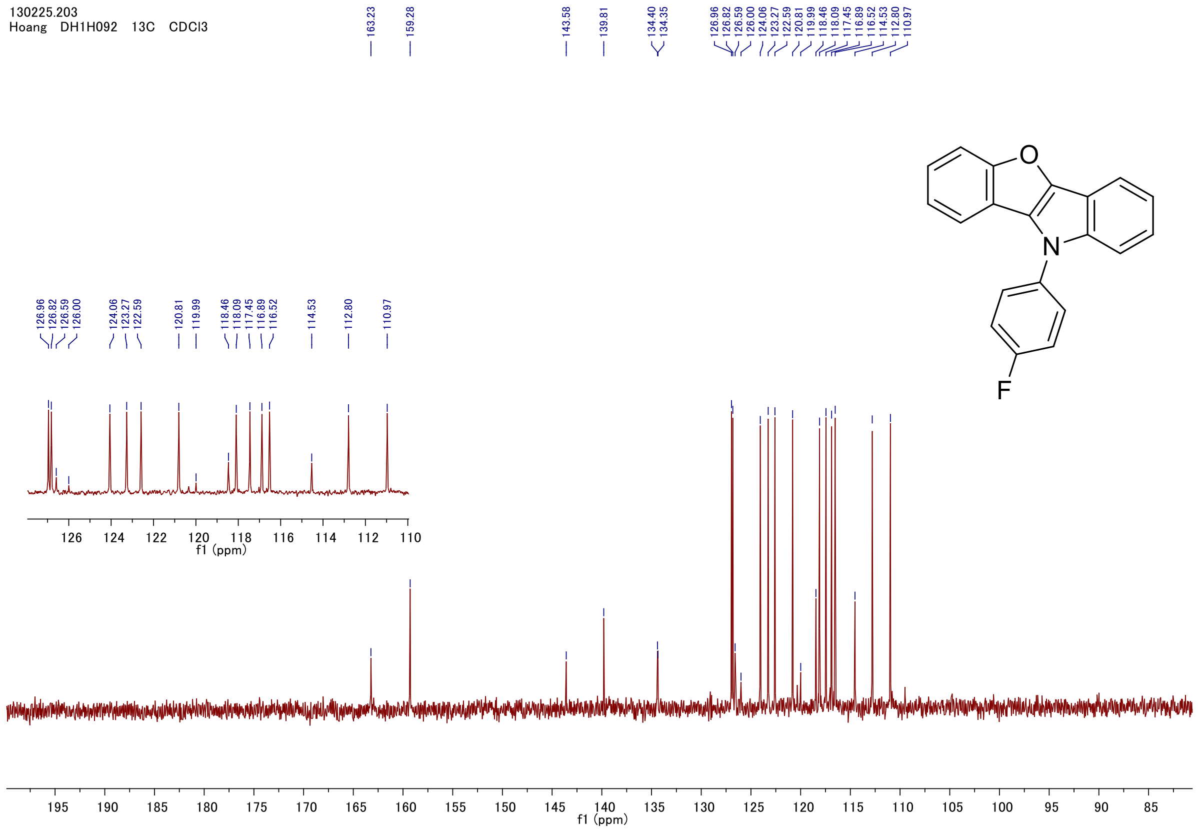

10-(3-(Trifluoromethyl)phenyl)-10*H*-benzofuro[3,2-*b*]indole (5d)

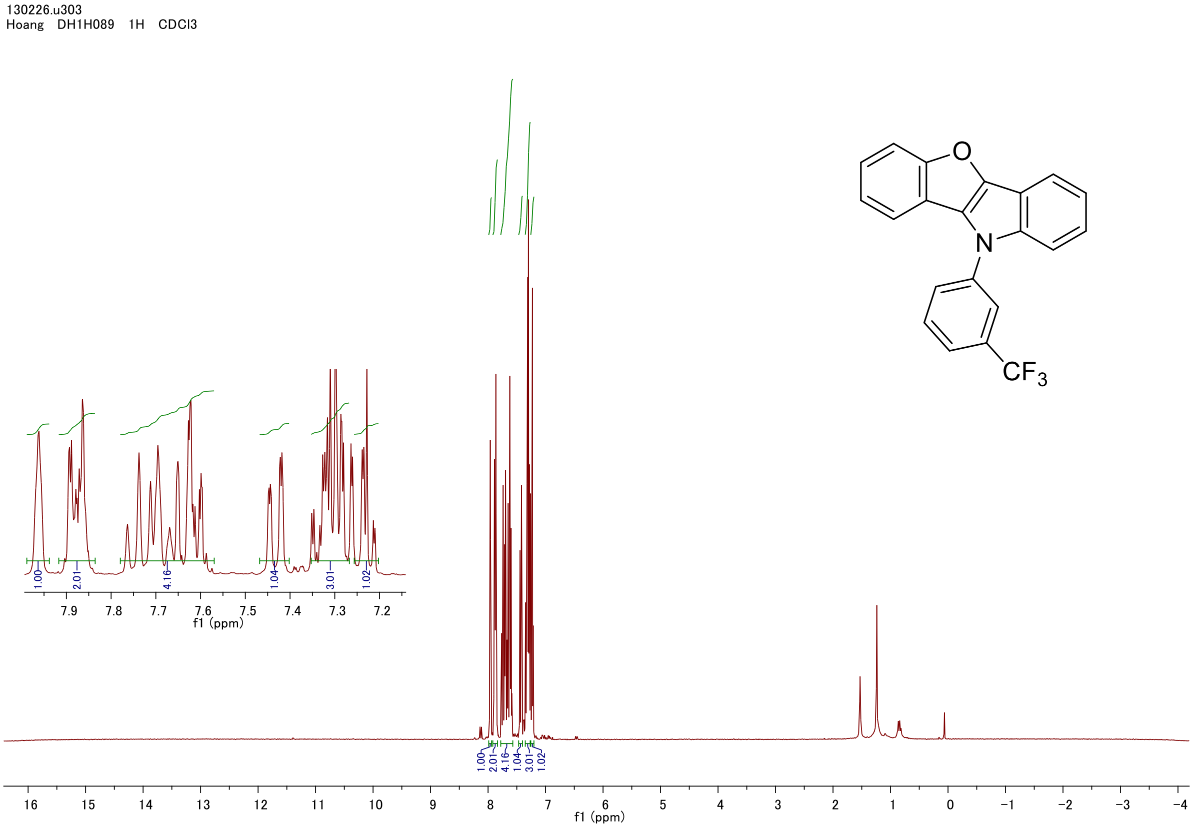

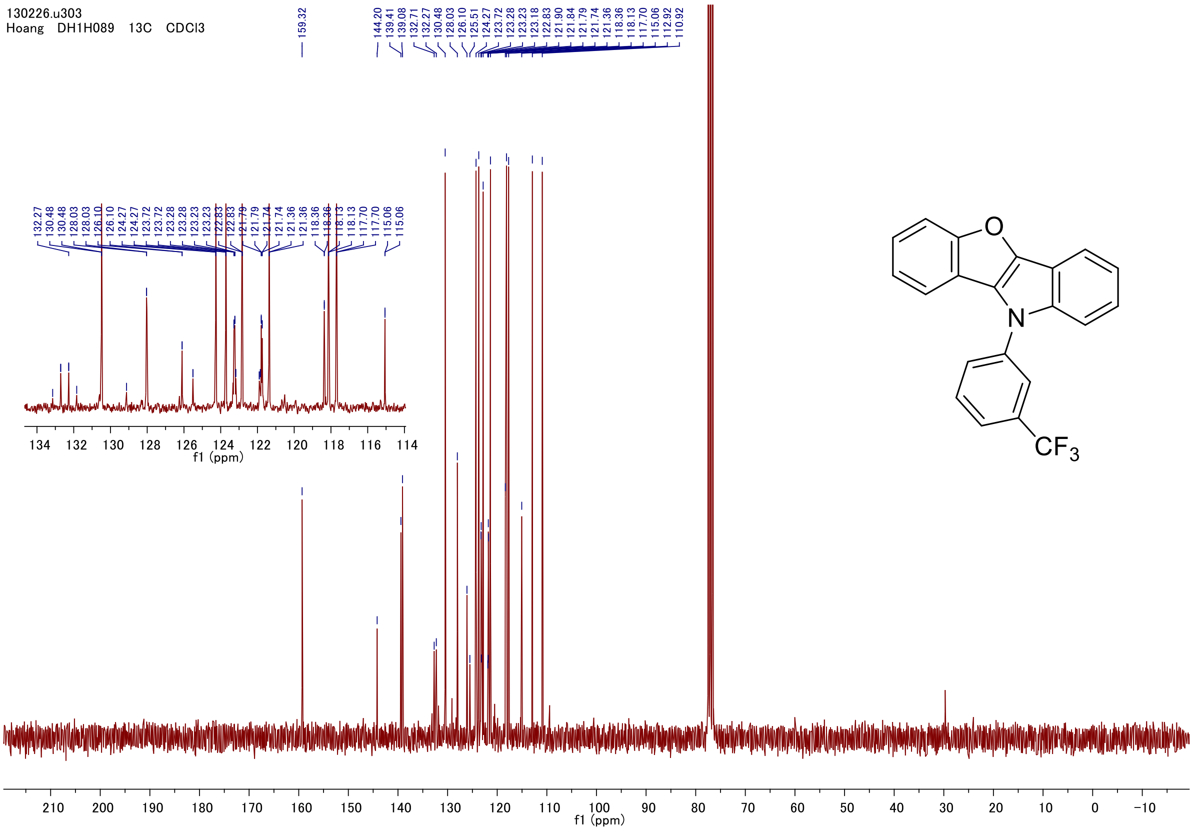

10-(4-Methoxyphenyl)-10*H*-benzofuro[3,2-*b*]indole (5e)

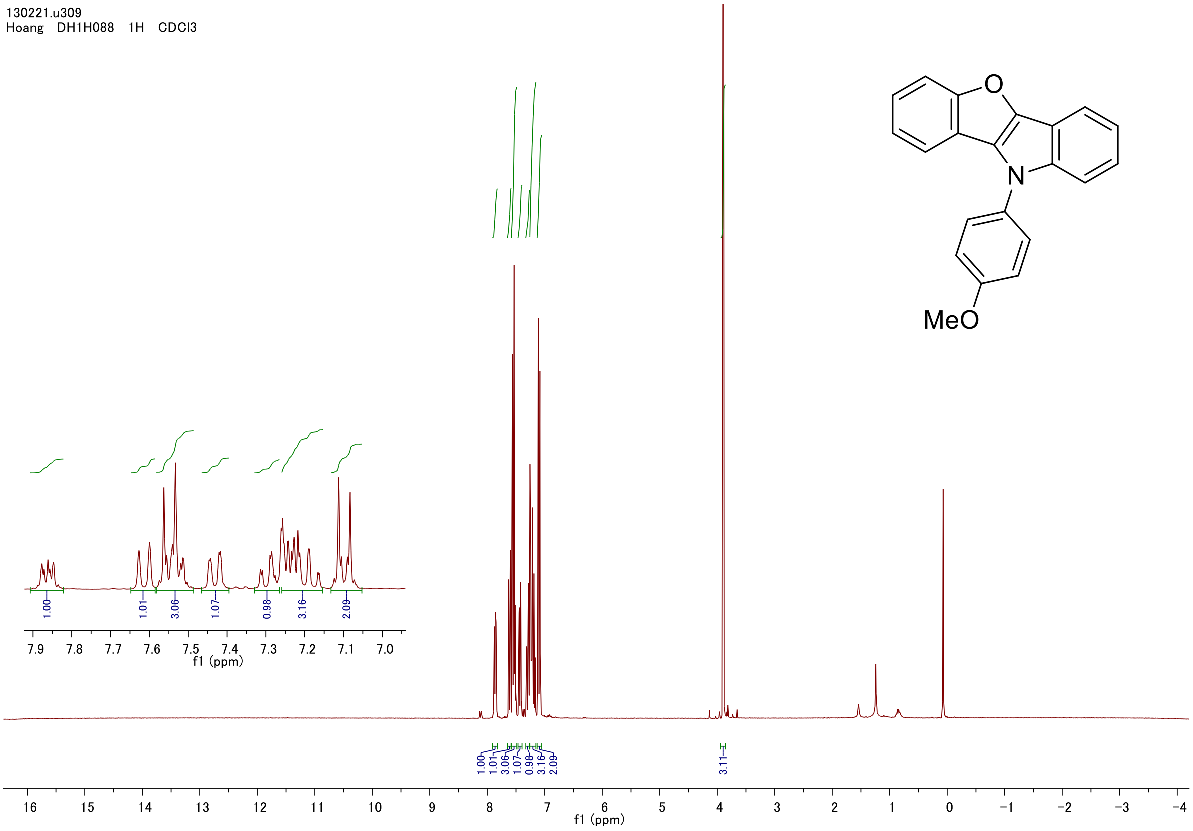

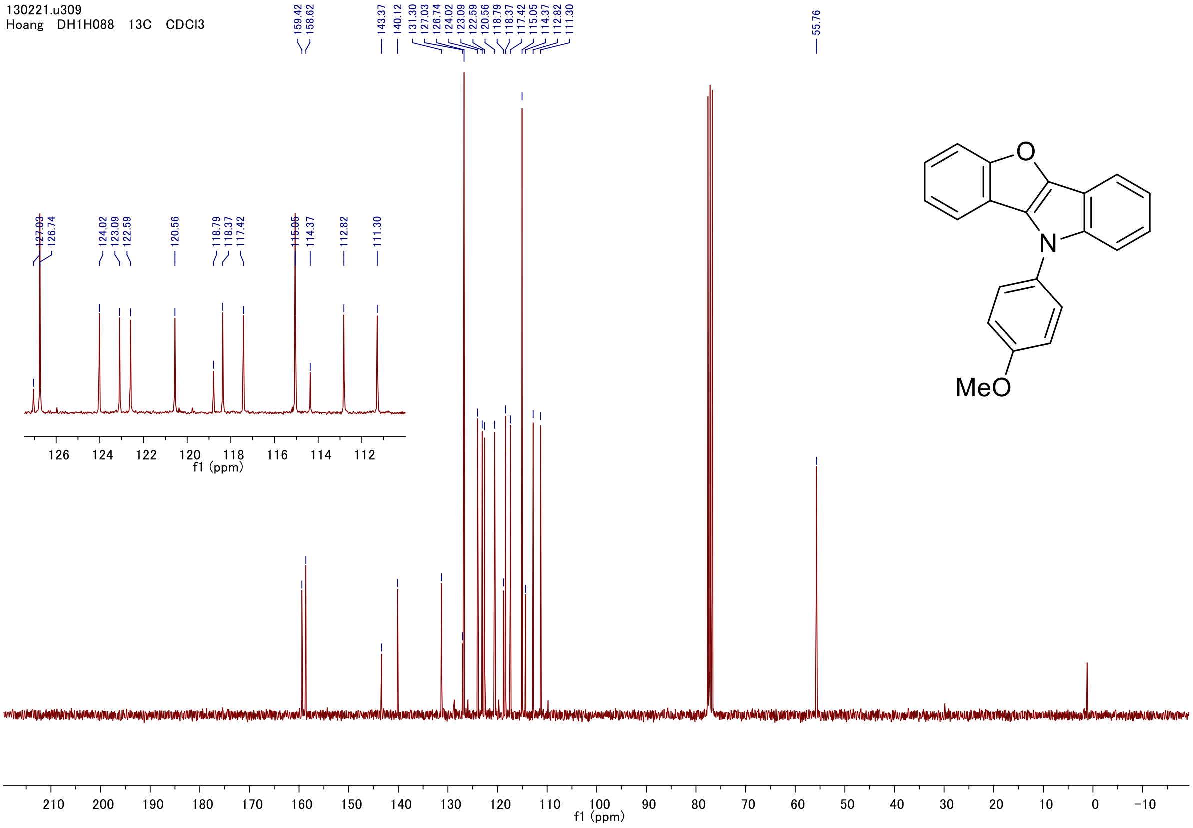

10-(3,4-Dimethoxyphenyl)-10*H*-benzofuro[3,2-*b*]indole (5f)

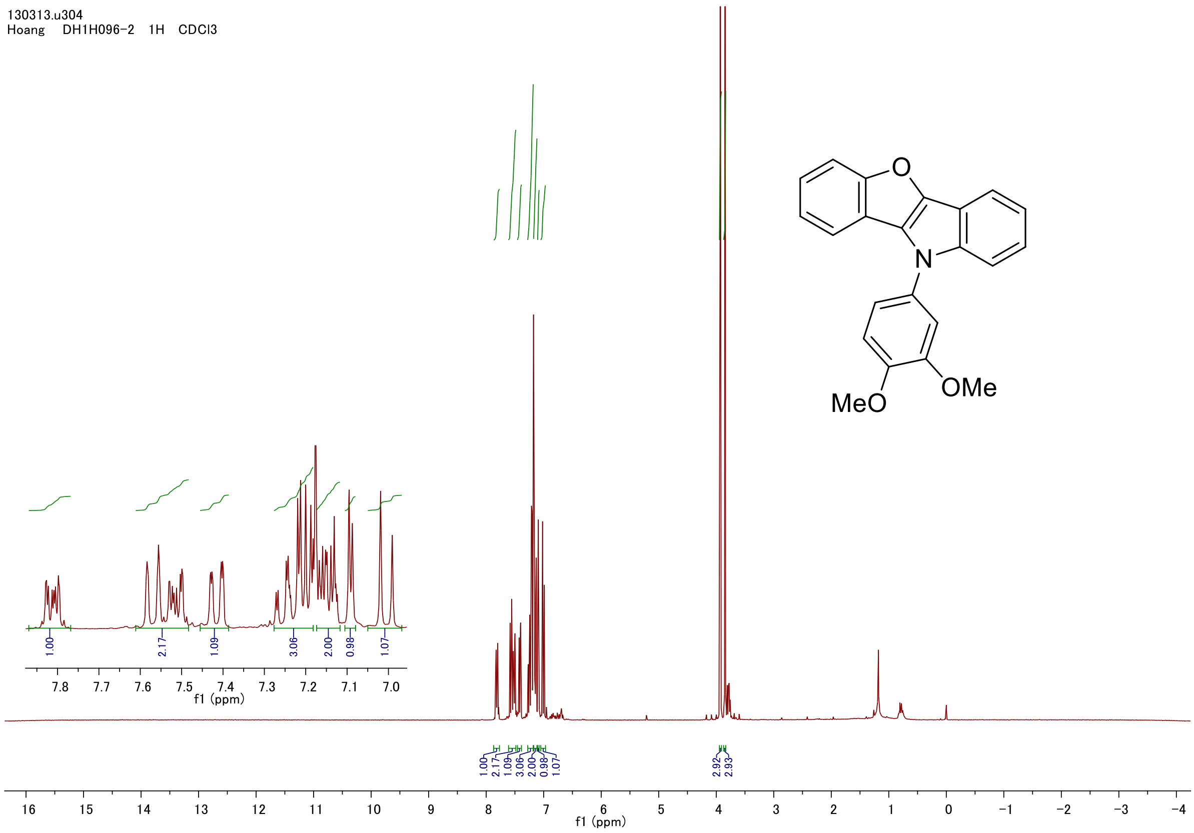

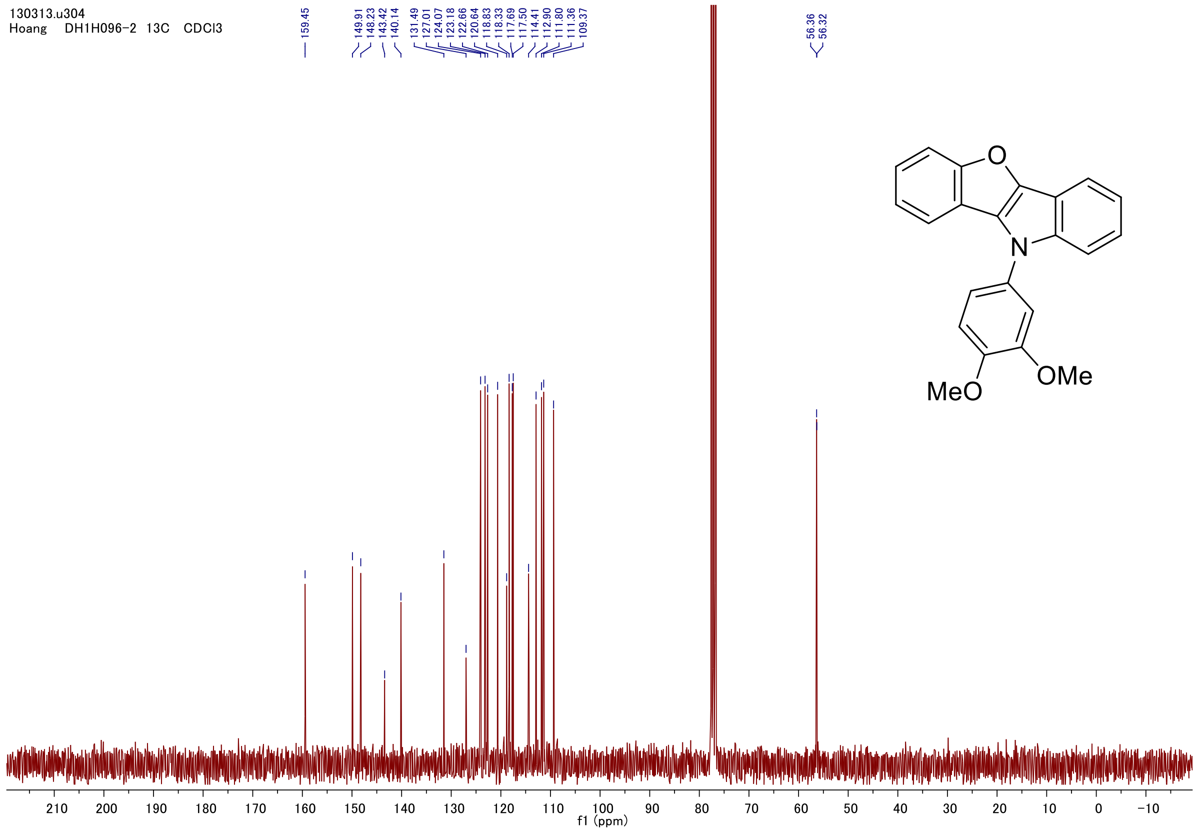

10-(4-(*tert*-Butyl)phenyl)-10*H*-benzofuro[3,2-*b*]indole (5g)

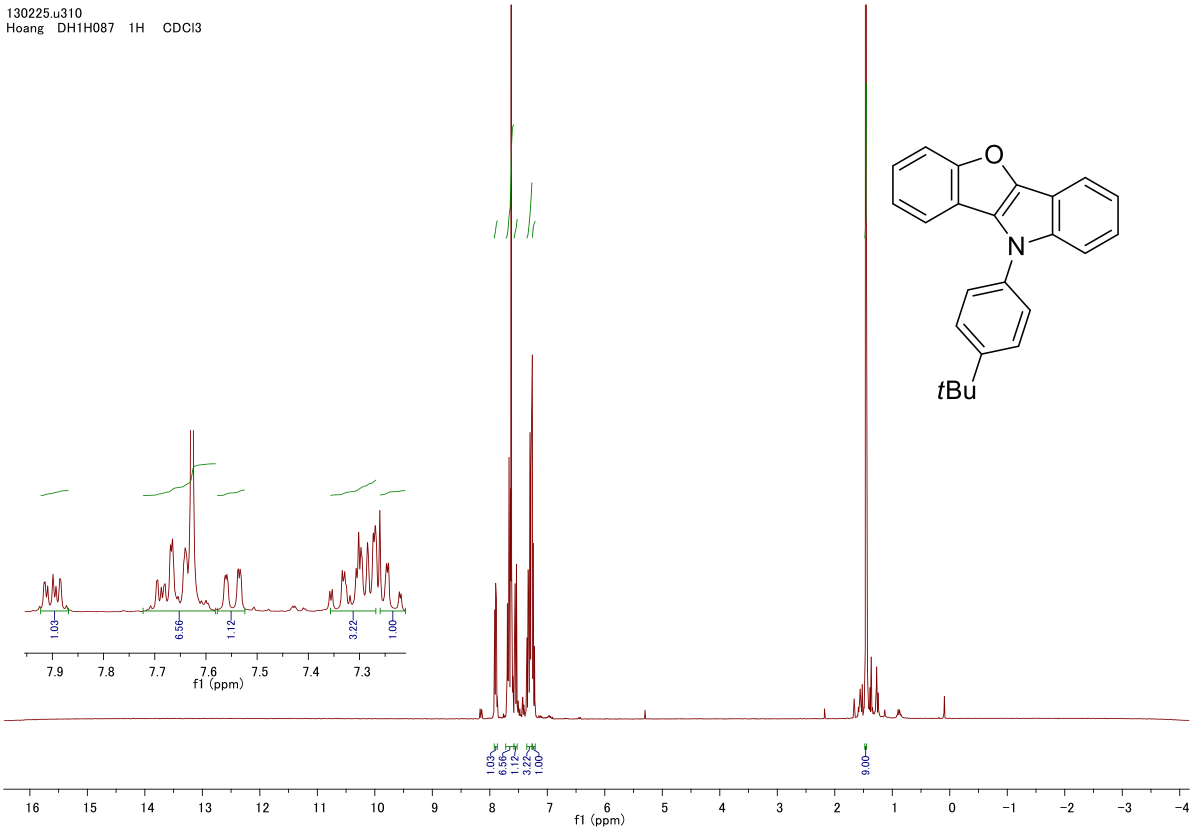

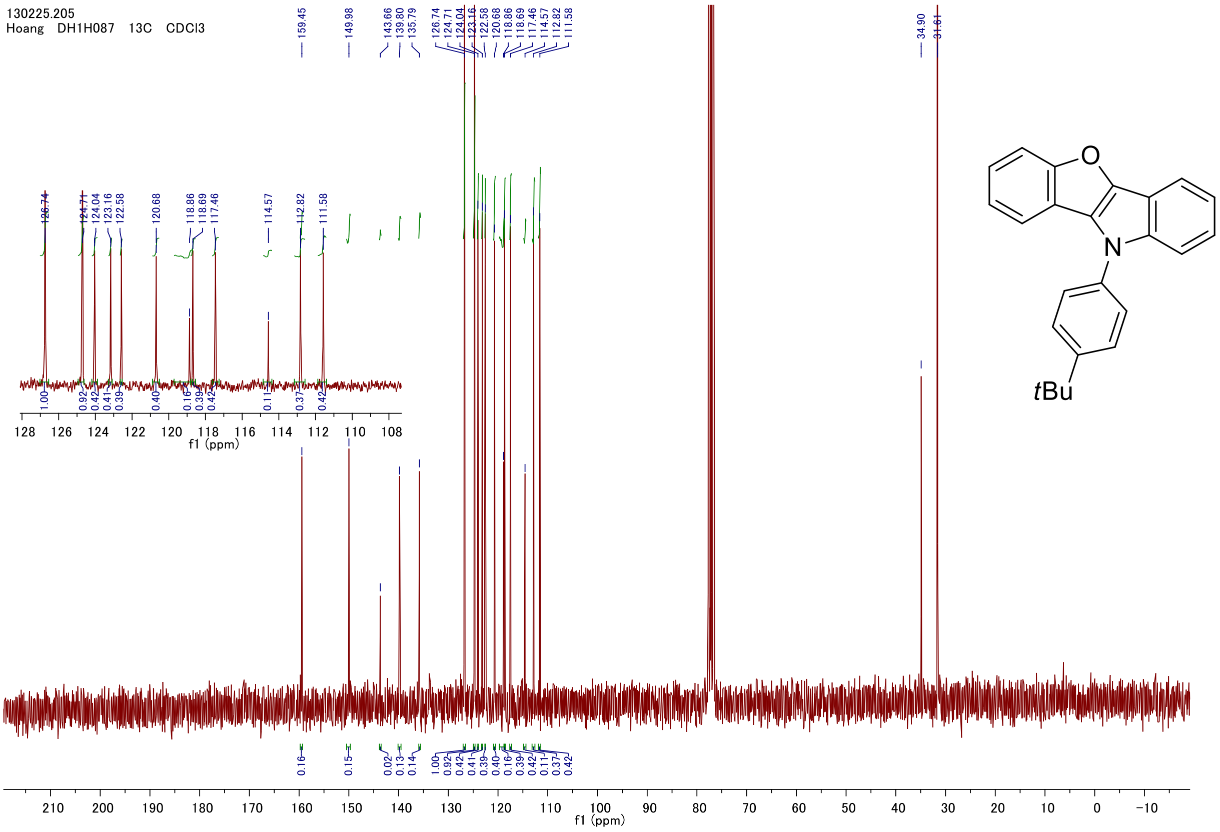

10-Benzyl-10*H*-benzofuro[3,2-*b*]indole (5h)

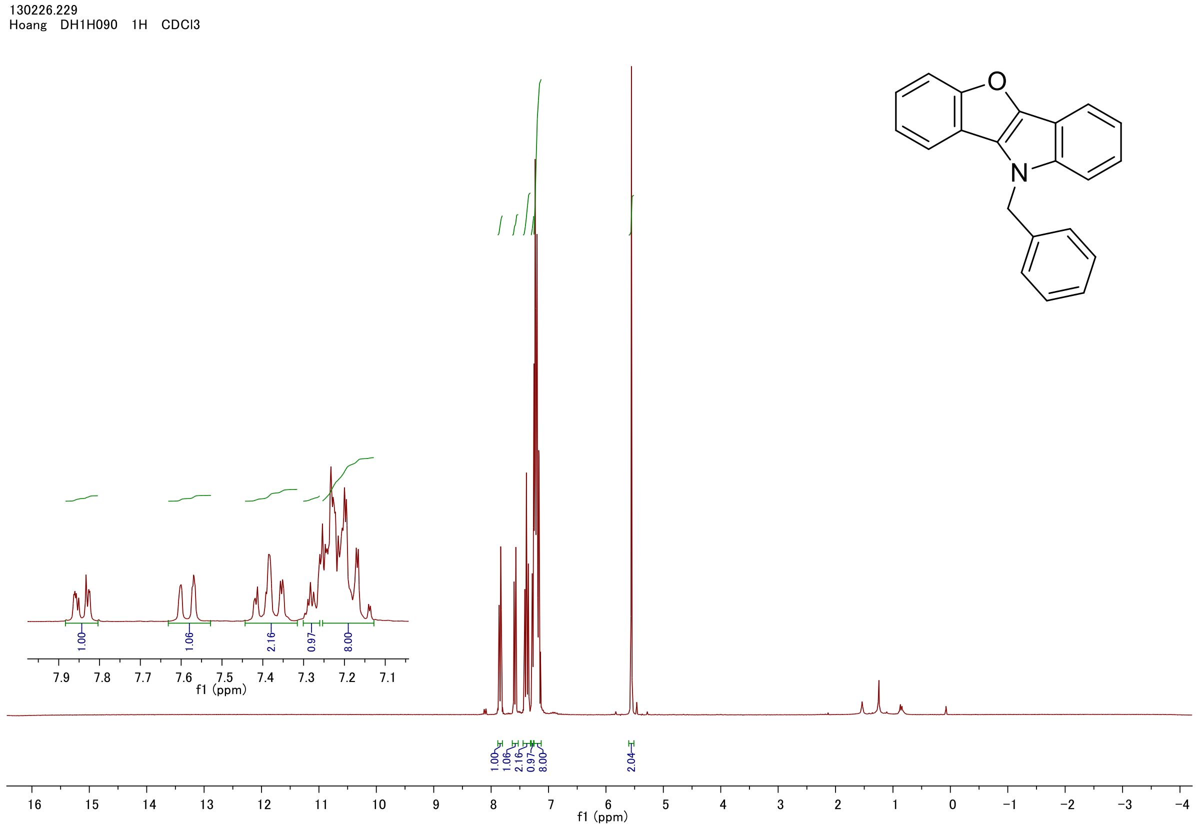

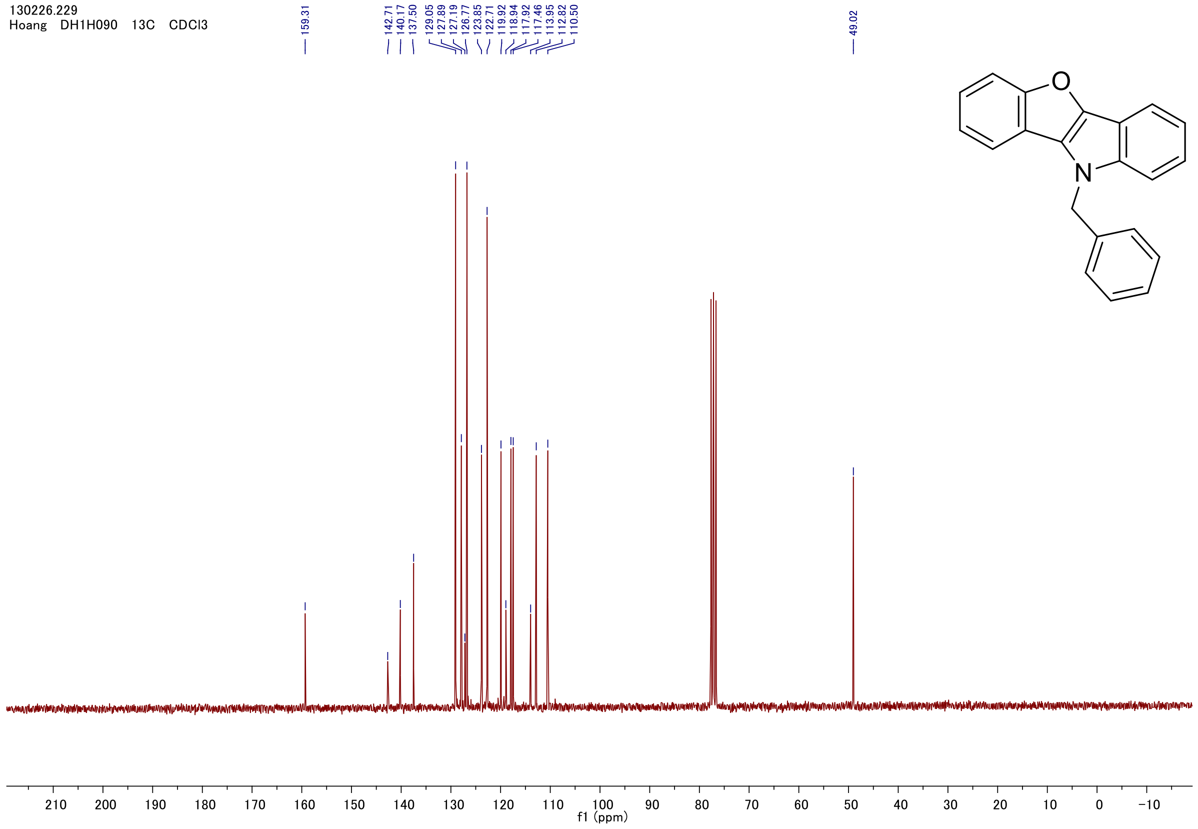

10-Heptyl-10*H*-benzofuro[3,2-*b*]indole (5i)

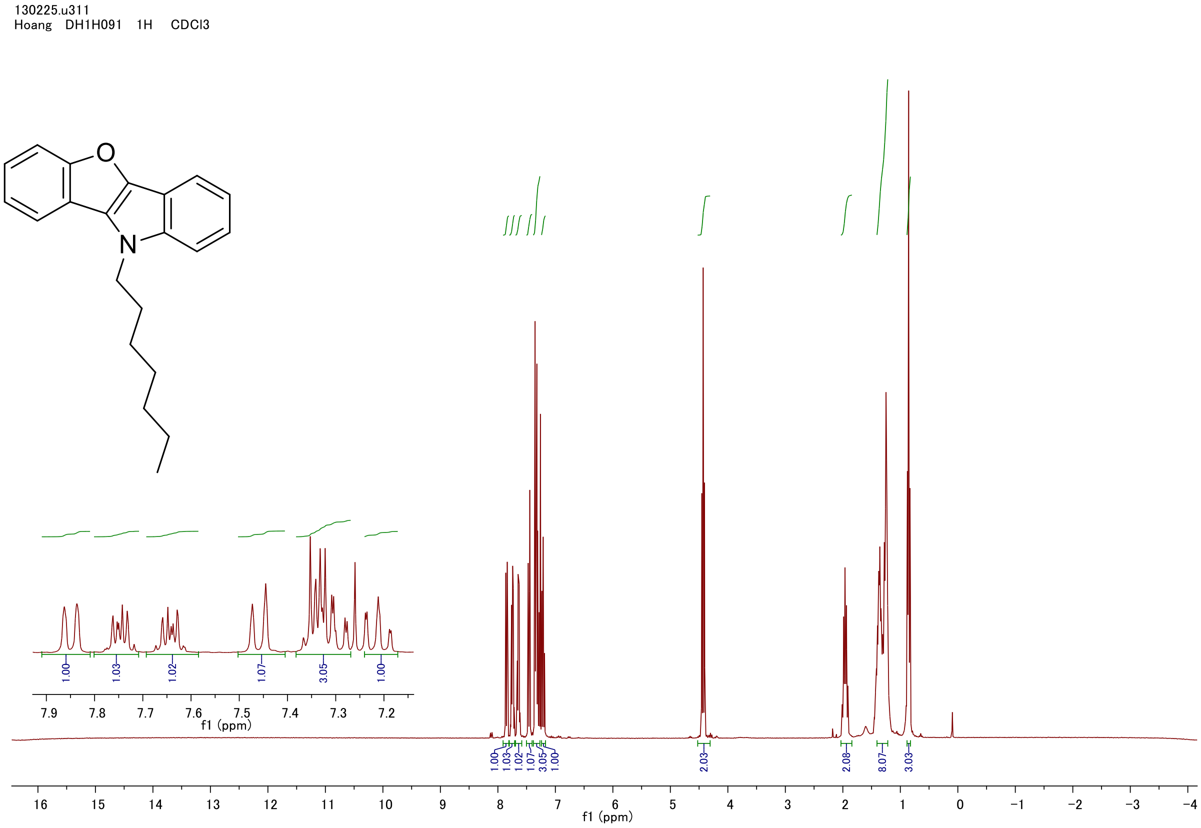

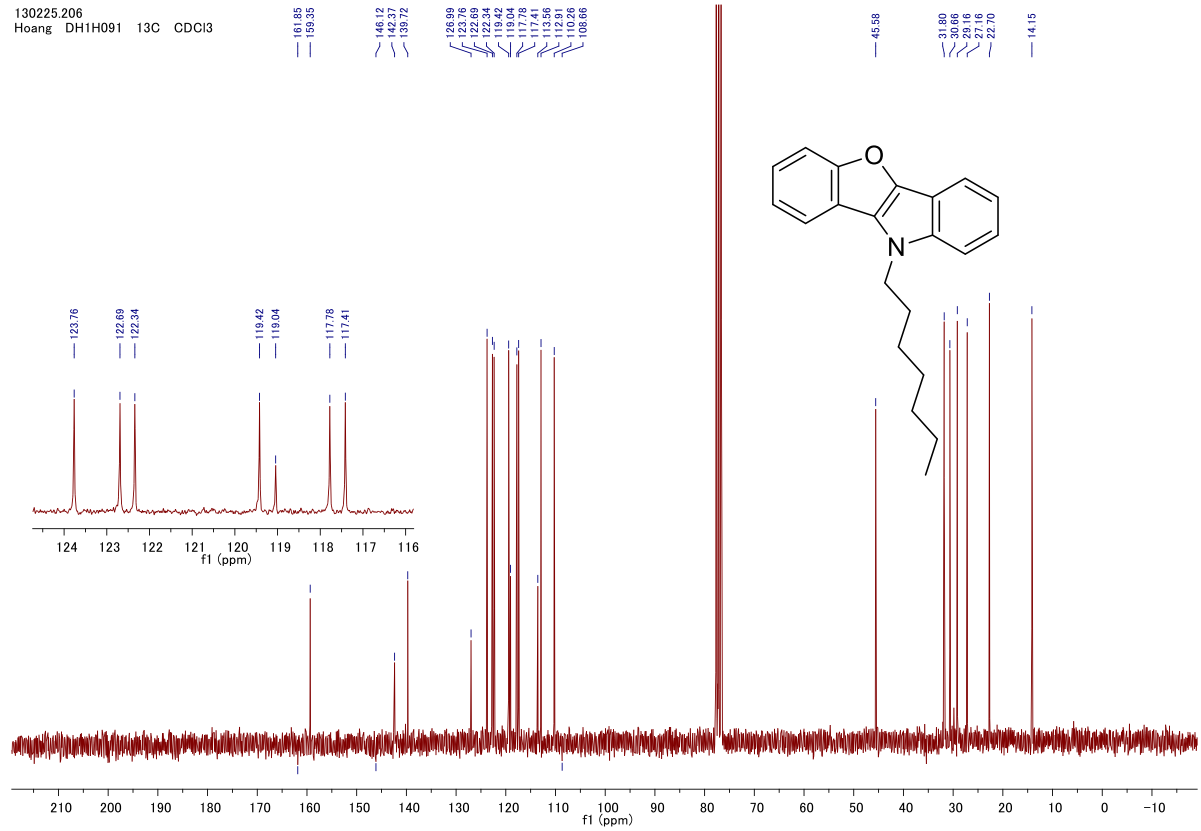

10-Cyclohexyl-10*H*-benzofuro[3,2-*b*]indole (5j)

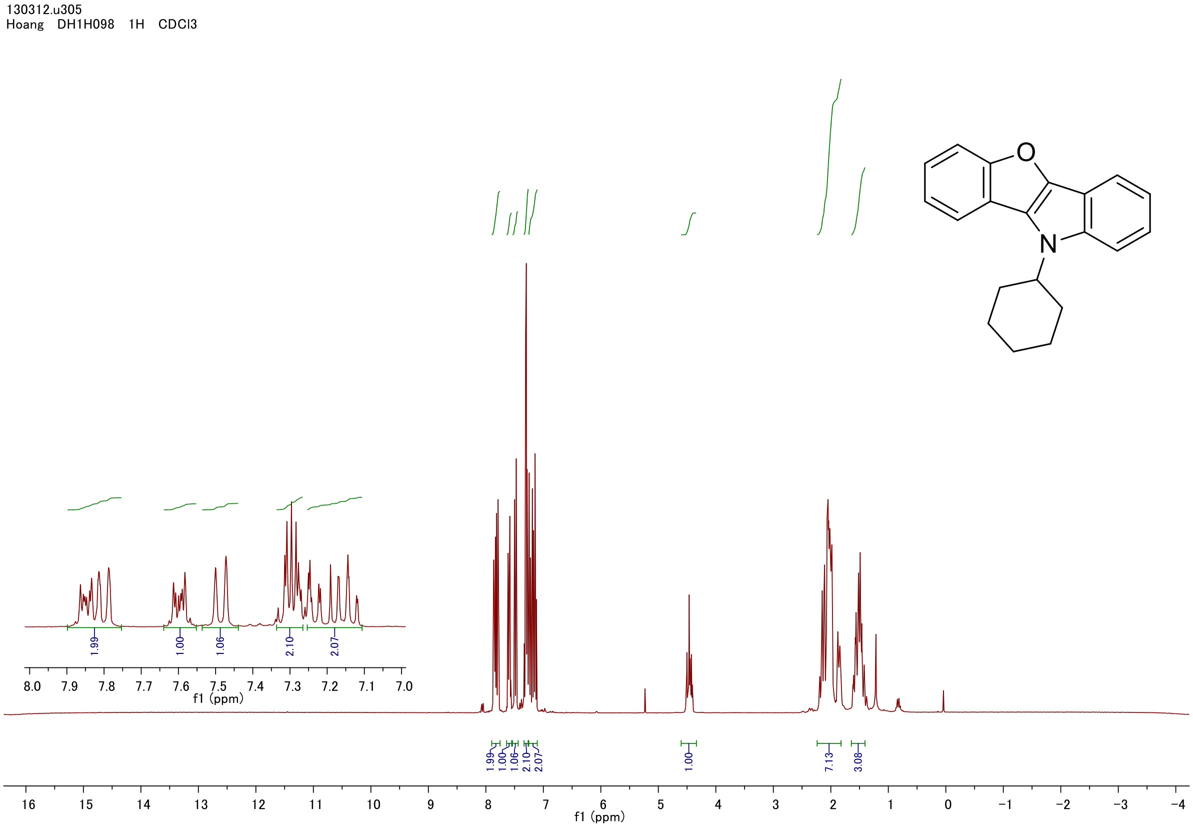

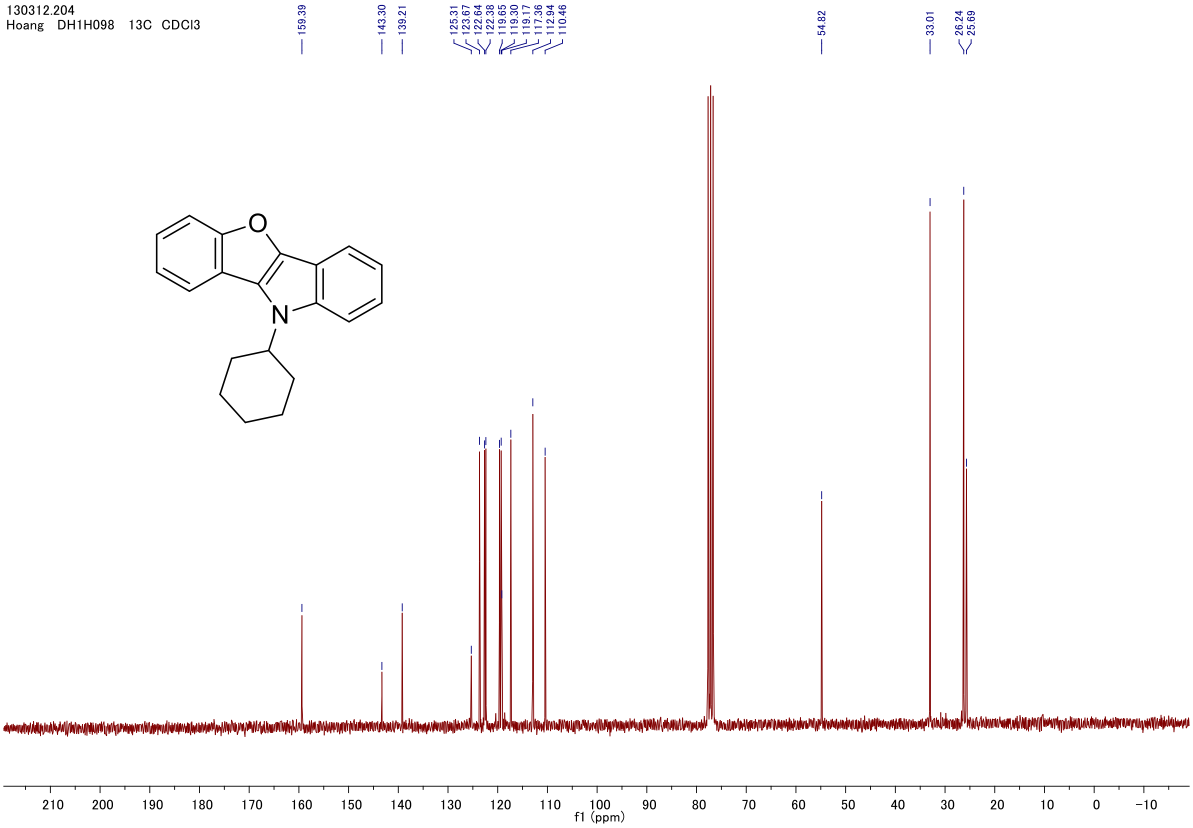

Biological protocols

Cell transfection with human NPPs. Analogous as previously described [1], COS-7 cells were transfected with plasmids expressing human NPPs ((NPP-1) [2] or (NPP-3) [3]) in 10 cm plates, by using Lipofectamine. The confluent cells were incubated for 5 h at 37 °C in DMEM/F-12 in the absence of fetal bovine serum and with 6 µg of plasmid DNA and 24 µL of lipofectamine reagent. Subsequently, the same volume of DMEM/F-12 containing 20% FBS was added to stop the transfection and cells were harvested 48–72 h later.

Preparation of membrane fractions. Analogous as previously described [1], the transfected cells were washed three times with Tris–saline buffer at 4 °C and then the cells were collected by scraping in the harvesting buffer (95 mM NaCl, 0.1 mM PMSF, and 45 mM Tris buffer, pH 7.5). Afterwards, the cells were washed twice by centrifugation at 300*g* for 5 min at 4 °C [4]. These cells were resuspended in the harvesting buffer containing 10 µg/mL aprotinin and then sonicated. Cellular and nuclear debris were discarded by 10 min centrifugation (300*g* at 4 °C). Glycerol (final concentration of 7.5%) was added to the resulting supernatant and all the samples were kept at −80 °C until used. Bradford microplate assay [5] was used for the estimation of protein concentration. Bovine serum albumin was used as a reference standard.

Protocol of nucleotide pyrophosphatase (h-NPP-1 & h-NPP-3) activity. Analogous as previously described [1], the conditions for the assay were optimized with the slight modifications in previously used spectrophotometric method [6]. The reaction was carried out in the assay buffer which contained 5 mM $MgCl_2$, 0.1 mM $ZnCl_2$, 25% glycerol and 50 mM tris-hydrochloride (pH: 9.5). Initial screening was performed at a concentration of 0.1 mM of the tested compounds. The total volume of 100 µL contained 70 µL of the assay buffer, 10 µL of tested compound (0.1 mM with final DMSO 1% (v/v)) and 10 µL of h-NPP-1 (27 ng of protein from COS cell lysate in assay buffer) or 10 µL of h-NPP-3 (25 ng of protein from COS cell lysate in assay buffer). The mixture was pre-incubated for 10 minutes at 37 °C and absorbance was measured at 405 nm as pre-read using microplate reader (BioTek FLx800, Instruments, Inc. USA). The reaction was then initiated by the addition of 10 µL of p-Nph-5-TMP substrate at a final concentration of 0.5 mM and the reaction mixture was incubated for 30 more min at 37 °C. The change in the absorbance was measured as after-read. The activity of each compound was compared with the reaction in absence of tested compounds/inhibitors. The compounds which exhibited over 50% inhibition of either the h-NPP-1 activity or h-NPP-3 activity were further evaluated for determination of IC_{50} values. For this purpose, their dose response curves were obtained by assaying each inhibitor concentration against both NPPs

using the above mentioned reaction conditions. All experiments were repeated three times in triplicate. The IC_{50} values, determined by the non-linear curve fitting program PRISM 5.0 (GraphPad, San Diego, California, USA).

References

1. Ngo, T. N.; Syeda, A. E.; Hung, T. Q.; Dang, T. T.; Iqbal, J.; Lecka, J.; Sévigny, J.; Langer, P. *Org. Biomol. Chem.* **2015**, *13*, 8277-8290.
2. Belli, S. I.; Goding, J. W. *Eur. J. Biochem.*, 1994, **226**, 433-443.
3. Jinhua, P.; Goding, J. W.; Nakamura, H.; Sano, K. *Genomics* 1997, **45**, 412-415.
4. Narisawa, S.; Harmey, D.; Yadav, M. C.; O'Neill, W. C.; Hoylaerts, M. F.; Millán, J. L. *J. Bone Miner. Res.* **2007**, *22*, 1700-1710.
5. Bradford, M. M. *Anal. Biochem.* **1976**, *72*, 248-254.
6. Tashfeen, A.; Shahid, H.; Najim, A. A. M.; Roberta, L.; Paolo. L. *Acta Pharm.* **2008**, *58*, 135-149.
